# Review on Predictive Models and Integration Strategies for Holistic Impact Assessment of Chemicals and Materials

**DOI:** 10.1021/acs.est.5c04489

**Published:** 2026-01-28

**Authors:** Angela Serra, Marcella Torres Maia, Periklis Tsiros, Vasileios Minadakis, Rafael Riudavets-Puig, Adrien Perello-y-bestard, Fotini Nikiforou, Achilleas Karakoltzidis, Emanuele Di Lieto, Alexandra Schaffert, Zeyad Al-Abdulraheem, Ishita Virmani, Olga Dziubaniuk, Sikri Karhukorpi, Joahim Dokler, Dimitrios Zouraris, Dimitris G. Mintis, Dimitra-Danai Varsou, Andreas Tsoumanis, Georgia Melagraki, Panagiotis Isigonis, Anastasios G. Papadiamantis, Marija Buljan, Anna Agalliadou, Laura-Jayne A. Ellis, Jacques-Aurélien Sergent, Matheus Alves Siqueira de Assunção, Diego Stéfani Teodoro Martinez, David Winkler, Seung-Geun Park, Seung Min Ha, Zayakhuu Gerelkhuu, Tae Hyun Yoon, Spyros Karakitsios, Dimosthenis A. Sarigiannis, Antreas Afantitis, Stefano Cucurachi, Tommaso Serchi, Antonino Marvuglia, Thomas Exner, Jaakko Siltaloppi, Martin Paparella, Willie Peijnenburg, Peter Wick, Iseult Lynch, Haralambos Sarimveis, Dario Greco

**Affiliations:** † Finnish Hub for Development and Validation of Integrated Approaches (FHAIVE), Faculty of Medicine and Health Technology, 7840 Tampere University, 33100 Tampere, Finland; ‡ Division of Pharmaceutical Biosciences, Faculty of Pharmacy, University of Helsinki, Helsinki 00790, Finland; § 68994 National Technical University of Athens, Zografou 15772, Greece; ∥ 111825 Swiss Federal Laboratories for Materials Science and Technology, Lerchenfeldstrasse 5, CH-9014 St. Gallen, Switzerland; ⊥ Institute of Environmental Sciences, 4496 Leiden University, P.O. Box 9518, 2300 RA Leiden, The Netherlands; # 37782 Aristotle University of Thessaloniki, Department of Chemical Engineering, Environmental Engineering Laboratory, University Campus, Thessaloniki 54124, Greece; 7 HERACLES Research Center on the Exposome and Health, Center for Interdisciplinary Research and Innovation, Balkan Center, Bldg. B, 10th km Thessaloniki − Thermi Road, Thessaloniki 57001, Greece; 8 Institute of Medical Biochemistry, 27280 Medical University Innsbruck, Innrain 80, Innsbruck 6020, Austria; 9 7840 Tampere University, Faculty of Management and Business, Industrial Engineering and Management Unit, PO Box 553, 33014 Tampere University, Finland; 10 Seven Past Nine d.o.o., Hribljane 10, 1380 Cerknica, Slovenia; 11 Entelos Institute, Nicosia 2102, Cyprus; 12 443801 NovaMechanics Ltd, Nicosia 1070, Cyprus; 13 NovaMechanics MIKE, Piraeus 18545, Greece; 14 Division of Physical Sciences and Applications, Hellenic Military Academy, 16672 Vari, Greece; 15 87145 Luxembourg Institute of Science and Technology (LIST), L-4362 Esch-sur-Alzette, Luxembourg; 16 Institute of Environmental Sciences, 4496 Leiden University, P.O. Box 9518, 2300 RA Leiden, The Netherlands; 17 National Institute for Public Health and the Environment (RIVM), Center for Safety Assessment of Substances and Products, 3721 MA Bilthoven, The Netherlands; 18 School of Geography, Earth, and Environmental Sciences, 1724 University of Birmingham, B15 2TT Birmingham, U.K.; 19 Centre for Environmental Research and Justice, 1724 University of Birmingham, Edgbaston, B15 2TT Birmingham, United Kingdom; 20 2056 Solvay SA, Toxicological and Environmental Risk Assessment Unit, Rue de Ransbeek 310, 1120 Bruxelles, Belgium; 21 306984 Brazilian Nanotechnology National Laboratory (LNNano), Brazilian Center for Research in Energy and Materials (CNPEM), Campinas, Sao Paulo 13083-970, Brazil; 22 Department of Biochemistry and Chemistry, La Trobe Institute for Molecular Science, La Trobe University, Bundoora, Victoria 3086, Australia; 23 Monash Institute of Pharmaceutical Sciences, Monash University, Parkville, Victoria 3052, Australia; 24 School of Pharmacy, University of Nottingham, Nottingham NG7 2RD, United Kingdom; 25 Department of Chemistry, College of Natural Sciences, 26716 Hanyang University, Seoul 04763, Korea; 26 Institute of Next Generation Material Design, 26716 Hanyang University, Seoul 04763, Korea; 27 Department of Pharmacy, Frederick University, Nicosia 1036, Cyprus

**Keywords:** impact assessment, Safe and Sustainable by Design (SSbD), predictive models, integrated impact assessment

## Abstract

Rapid innovation in chemicals and materials calls for innovative integrated approaches that can assess their impacts across different areas. The Safe and Sustainable-by-Design (SSbD) framework, developed by the European Commission’s Joint Research Centre (JRC), offers a comprehensive approach with which to evaluate the safety and sustainability of chemicals and materials across their lifecycle. While SSbD uses various modeling approaches to assess impacts on human health, the environment, and socioeconomic factors, these are often applied independently, hindering a holistic understanding of the complex interactions between these factors and thus the simultaneous optimization of function, cost, safety and sustainability. This review describes existing predictive models and available strategies for their integration to facilitate more comprehensive and holistic chemical and material impact assessments. Specifically, we examine three model integration strategies: consensus integration that combines model predictions for the same impact categories, weighted aggregation that combines different scores in a unified one, and pipeline integration that links models sequentially to create a more unified assessment. Furthermore, we address key concepts related to the uncertainty of model predictions and the applicability domain of models, highlighting how these evolve in integrated frameworks. Insights into the applications of these integration strategies and challenges will allow a more accurate, coherent, and sustainable approach to chemical and material safety and sustainability assessments.

## Graphical abstract

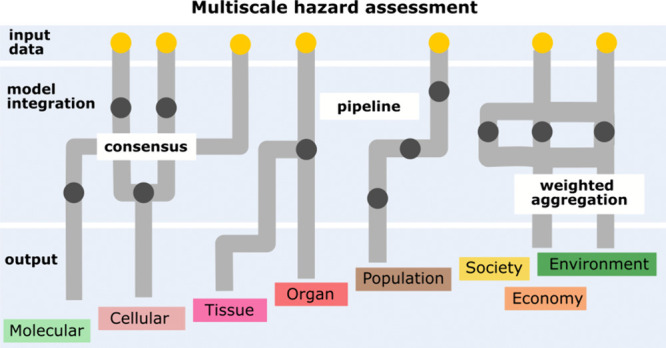

## Introduction

The rapid emergence of novel chemicals and advanced materials has prompted the need for new approaches to their impact assessment. The focus is on the development of predictive integrated approaches that can address multiple safety and sustainability dimensions. This evolution has led to the development of the Safe and Sustainable-by-Design (SSbD) framework,^1,2^ established by the European Commission’s Joint Research Centre (EC JRC). The framework evaluates the safety and sustainability of chemicals and materials by examining their impacts on human health, the environment, and socioeconomic factors,^1,2^ thereby offering a more holistic approach to managing chemicals across their lifecycle. The framework operates in two main phases, the (re)­design and the assessment phases, that are applied iteratively. The assessment phase contains five steps: hazard assessment (of the raw materials and production processes); human health and safety during production and processing; human health and environmental aspects in the final application; environmental sustainability; and socioeconomic analysis.^3^

To support these steps, a range of existing modeling tools are used by stakeholders to enable and facilitate robust impact assessment of chemicals and materials. This review explores and describes the available models suitable for impact assessment under the SSbD framework.

These include AOP (Adverse Outcome Pathways) based models that offer a structured framework to link molecular-level event to adverse health or ecological outcomes, quantitative structure–activity relationship (QSAR) models for hazard prediction, dose–response and toxicokinetic models for internal exposure estimation, toxicogenomics based approaches that provide mechanistic insights into biological perturbations, read-across methods that enable data extrapolation across similar substances. Beyond safety assessment models, life cycle assessment (LCA) and social LCA (S-LCA) models are also increasingly used to evaluate the broader sustainability impacts of chemicals and materials. Moreover, exposure and environmental fate models further contribute to the estimation of distribution and persistence of substances across environmental compartments. More recently, digital twins have also emerged as promising tools for real-time simulation and optimization in the SSbD context.

These tools, however, are often used independently, addressing individual aspects of safety or sustainability without considering their interrelationships. This fragmented approach hinders comprehensive impact assessment, as the interplay between different impact categories (e.g., climate change, acidification, resource depletion, toxicity, and ozone depletion) is not captured. Therefore, it is beneficial to integrate these diverse models into a cohesive framework, enabling a more holistic evaluation involving complex interactions across the environmental, health, and socioeconomic dimensions.^4^

In this review, we identified three integration approaches: consensus integration, weighted aggregation and pipeline integration. Consensus integration builds a consensus prediction of multiple models for the same impact category, weighted aggregation combines different scores for the same impacts into a unified score, and pipeline integration concatenates multiple models with shared input/output values to perform an integrated assessment. Moreover, we also address the important concepts of uncertainty propagation and applicability domains in the different integration strategies, as a key aspect of building user (industry and regulator) confidence in the operationalization of the SSbD framework. Examples of applications of individual models and model integration strategies are described in this paper, for advanced materials, such as engineered nanomaterials (ENMs), and polyfluoroalkyl substances (PFAS). ENMs represent an advanced family of materials with considerable technological potential in many industrial sectors, yet their long-term environmental and health impacts remain uncertain.^5^ PFAS, by contrast, exemplify a group of chemicals with well-established persistence and toxicity concerns, yet they are still widely used in industry.^6^ Finally, we present the INSIGHT EU project strategy as an illustrative example to contextualize the concepts discussed in this review and to demonstrate how the model integration strategies can be embedded within a unified framework. This framework exemplifies how these approaches can be operationalized to enhance mechanistic, multiscale, and multidomain impact assessment for chemicals and materials.^4^

## Models for Impact Assessment and SSbD of Chemicals and Materials

A suite of specialized models is essential for integrated impact assessment and SSbD of chemicals and materials (Figure 1, Table S1). Table S1 summarizes the available tools, the SSbD aspects or characteristics they address, and their limitations. These currently available models include Adverse Outcome Pathways (AOP) based models, quantitative structure activity relationship (QSAR) models, Physiologically Based Kinetics (PBK) models, toxicogenomics (TGx)-based models, dose–response models to derive Point of Departure (PoD) and read-across and grouping approaches. Furthermore, exposure and environmental release and fate models enable risk assessment by comparing reference values with exposure estimates. Finally, models for life cycle assessment (LCA), social-LCA (S-LCA), and life cycle costing (LCC) support the integration of toxicological risk estimates into a broader sustainability assessment. The next sections describe each of these model types, provide examples of their use in the literature along with discussion on possible limitations associated with these modeling approaches.

**Figure 1:**
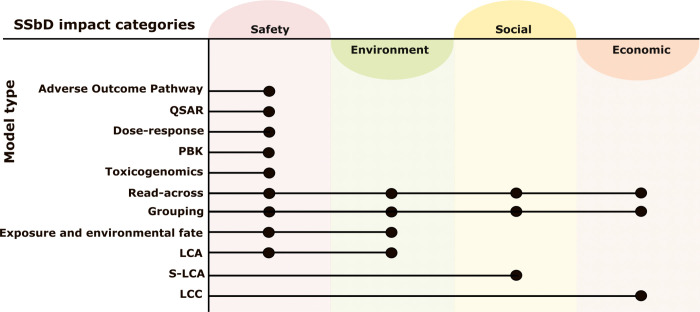
Mapping of existing models to various impact categories in SSbD (human and environmental safety; environment, social, and economic impacts) that can be integrated for a comprehensive impact assessment.

## Adverse Outcome Pathway-Based Models

Traditional toxicological risk assessment relies on resource-intensive *in vivo* studies, which focus on apical end points and often fail to provide insights into underlying toxicity mechanisms. This makes it difficult to understand and anticipate hazardous chemical properties and proactively modify them within SSbD. In contrast, Adverse Outcome Pathways (AOPs) provide a structured framework that describes the mechanistic progression from a molecular initiating event (MIE) through key biological events (KEs) to an adverse outcome (AO).^7^ They operate across multiple biological levels and time scales, integrating molecular perturbations with cellular, tissue, organismal, and population-level effects. By mapping outputs from new approach methodologies (NAMs), including omics, high-content *in vitro* assays, and in silico models, onto KEs and key event relationships (KERs), AOPs can enable early, mechanism-based hazard identification and provide traceable mechanistic anchors for design decisions, and reduce the need for *in vivo* testing. AOPs supply the causal mechanistic context and traceability required to interpret nonanimal and mechanistic data, prioritize candidates, and align minimal yet informative testing strategies with explicit disease end points.

Importantly, AOPs contribute to the One Health dimension of SSbD by offering a framework for cross-species and ecosystem-wide integration of chemical risks. One Health is an emerging approach that emphasizes the interconnectedness of health of humans, animals, plants and the environment.^8^ Since many molecular and cellular toxicity mechanisms are evolutionarily conserved, AOPs can support the mechanistic extrapolation of toxic effects across different species, making them useful for both human health and environmental safety assessments.^8^ Omics-based approaches could put the One Health application of AOPs in practice by identifying evolutionarily conserved molecular responses to toxic exposures, as shown by a recent analysis of transcriptomics data from various ENM nanomaterial exposure studies, which demonstrated that immune-related pathways are consistently deregulated across multiple species, even in nonimmune cells.^9^ Additionally, omics data can refine AOP-based hazard assessment by increasing mechanistic resolution, identifying biomarkers, and improving pathway completeness. Saarimäki et al. systematically curated gene-KE-AOP associations, demonstrating that integrating omics-based evidence allows for a more detailed understanding of key biological events and their relationships within the AOP, improves the identification of potential AOs, and can guide the development of mechanistically informed assays for chemical hazard characterization.^10^ del Giudice et al. further propose that embedding those omics-AOP maps into network-based approaches can improve predictive accuracy by leveraging gene–gene relationships and system topology.^11^

Where quantitative evidence is available, quantitative AOPs (qAOPs) extend qualitative AOPs with dose–response and temporal structure and with explicit representations of uncertainty. In practice, this entails parametrizing KERs with functions that relate the magnitude and time course of an upstream KE to a downstream KE or AO, using data from *in vitro* concentration–response experiments, time-series omics, and, where relevant, PBK models to translate external doses into target-site concentrations. qAOPs can employ computational modeling approaches, such as dose–response relationships, differential equations (DE), and Bayesian network modeling (BNN), to estimate the likelihood, timing, and severity of how perturbations at early KEs propagate through the pathway. For example, Jeong et al. (2018) used BNN to link oxidative stress to reproductive effects in C. elegans exposed to silver nanoparticles.^12^ In this way, qAOPs support SSbD by enabling quantitative ranking of design alternatives and a more refined prioritization of substances with a lower probability of adverse effects.

Even as AOPs advance mechanism-based assessment, limitations in coverage and quantification constrain their application in SSbD contexts. Many pathways remain largely qualitative, and existing coverage does not yet span all relevant species, life stages, or SSbD-relevant outcomes. In addition, the multiscale biological data required to support full pathway resolution are often scarce in the very early stages of innovation. These constraints, however, do not eliminate practical utility. AOP weight-of-evidence assessment while recognizing uncertainty can still inform early hazard identification, and even partial pathway information can provide valuable mechanistic guidance to support prioritization and design refinement. Additionally, to fully leverage AOP-enabled mechanistic assessment processes within SSbD, hazard assessment must shift toward mechanism-based evaluation and a new NAM-based classification system (see section “Redefining Toxicology: Advancing NAMs and Computational Models for Sustainable Risk Assessment”).

## Quantitative Structure–Activity Relationship (QSAR) Models

While AOP-based models provide a mechanistic framework for linking MIE to AO, QSAR models can complement this by enabling predictive hazard assessment directly from chemical characteristics. QSAR models use machine learning (ML) or statistical methods to predict end points relevant for human and environmental health impacts, based on the molecular structure and/or properties of the chemicals or materials of interest.^13,14^ QSAR models are essential for initial screening and subsequent assessment of new chemicals, particularly those with limited data. They support prioritization and help reduce the need for animal testing.^15^ Regulatory QSAR tools applicable to impact assessment include the OECD QSAR Toolbox,^16^ VEGA,^17^ and the Danish (Q)­SAR database.^18^ These tools have models to predict hazards such as mutagenicity, skin irritation and corrosion, eye damage, endocrine disruption, chronic and acute environmental toxicity, carcinogenicity, and ecotoxicity providing a firm basis for assessing chemical safety. The applicability domain (AD) defines the chemical and structural boundaries for reliable predictions of an individual model.^19^ QSAR models should be documented using the (Q)­SAR Model Reporting Format (QMRF) to ensure transparency, reproducibility, and regulatory acceptance,^20^ following the validation principles defined by the Organisation for Economic Cooperation and Development (OECD).

QSAR models are widely used to study PFAS, a group of over 4,000 synthetic chemicals valued for their water- and grease-resistant properties. However, their persistence, bioaccumulation, and toxicity (PBT) pose significant environmental and health concerns.^21–23^ The limited availability of experimental data for many PFAS compounds underscores the need for predictive models to aid regulatory assessment. For example, Gallagher et al. integrates computational techniques, including QSAR modeling, molecular docking, and read-across analysis, to explore the interactions of PFAS with human serum albumin.^24^ Moreover, research by da Silvia and de Melo leverages multivariate QSAR modeling to predict the toxicity of PFAS in rats and mice via oral and inhalation exposure.^25^ Sosnowka et al. reviewed 38 QSAR models for PFAS properties, assessing their scientific validity and reproducibility using QMRF reports, finding 22 models valid but many lacking regulatory suitability.^26^

QSAR modeling extends beyond small molecules to advanced materials, like ENMs, with approaches known as nano-QSAR that link physicochemical properties of ENMs to their adverse effects. For example, Puzyn et al. developed a model to predict the toxicity of metal oxide-based ENMs to the bacteria, *Escherichia coli* *.* ^27^ Huang et al. devised a model to predict inflammatory potential of metal oxide ENMs by assessing the release of proinflammatory cytokine interleukin (IL)-1 beta as the end point.^28^ To improve nanoscale structure–activity relationship (nano-QSAR) model, Ha et al.,^29^ and Choi et al.,^30^ developed nano-SAR models for metal oxide nanoparticles, integrating physicochemical and toxicological attributes, while incorporating data gap-filling and PChem score-based screening to enhance data set quality, highlighting the importance of preprocessing and quality control in nano-QSAR modeling. Additionally, Choi et al., introduced a quasi-QSAR model using quasi-SMILES encoding and hierarchical clustering analysis (HCA) to improve cell viability predictions for human lung and skin cells exposed to metal oxide nanoparticles.^31^ Trinh et al., developed a nanoSAR classification model for metallic nanoparticles, addressing key challenges such as data heterogeneity and quality gaps in publicly available nanotoxicity data sets.^32^ Qi and Wang developed a model to predict the oxidative stress potential of both individual and combined CNM in the algae, *S. obliquus*.^33^ User friendly implementation of nano-QSAR models is also offered on the Enalos Cloud platform for the prediction of biological and toxicological profiles of ENMs, such as multiwalled carbon nanotubes (MWCNTs).^34^

Another aspect addressed in QSAR modeling is the incorporation of mechanistic insights, such as AOPs, to significantly enhance predictive accuracy and regulatory relevance. In this context, Jagiello et al. introduced an AOP-informed QSAR approach focused on ENMs to predict transcriptomic changes associated with lung tissue inflammation, an event in the lung fibrosis AOP:173 on the AOP-Wiki (https://aopwiki.org/).^35^ The authors have anchored point of departure (PoD) values to structural properties of multiwalled carbon nanotubes (MWCNT) and found that the aspect ratio of the MWCNTs could predict the observed biological responses. Moreover, Seo et al. developed QSAR models to predict biocidal mixture effects leading to pulmonary fibrosis through AOP 347, implementing two MIEs: TLR4 activation and PPAR-γ gamma inactivation. These models demonstrated superior predictive accuracy compared to energy-based methods (e.g., MD) when validated against human lung cell assays, exemplifying how AOP frameworks can guide mixture toxicity modeling for pulmonary fibrosis prediction.^36^ In another study, Gadaleta et al. developed comprehensive QSAR models predicting chemical bioactivity toward protein targets associated with MIEs related to liver steatosis, cholestasis, nephrotoxicity, and cognitive dysfunction. These types of QSAR based MIE predictions may provide relevant information on assay prioritization among a battery of *in vitro tests* available for assessing chemical toxicity.^37^ Similarly, Viganò et al. employed AI-based QSAR models within AOP framework to evaluate cardiotoxicity, focusing on MIEs leading to cardiotoxicity-related AOs, such as mitochondrial complex inhibition, oxidative stress, and mitochondrial dysfunction.^38^

However, reliance on generic physicochemical descriptors (e.g., molecular weight, dipole moment, surface area) in classical QSARs models often limits mechanistic reliability when applied to new compounds outside the training set (i.e., external testing). By contrast, physics-based approaches, including quantum-mechanics (QM)-informed models have demonstrated greater robustness and mechanistic transparency across several end points. For example, pharmaceutical N-nitrosamine impurities have been investigated as potential carcinogens, where QM-derived bioactivation/α-hydroxylation metrics and related electronic descriptors stratify carcinogenic potency and outperform generic category rules.^39,40^ For skin sensitization, mechanistic domain models augmented with QM reactivity descriptors (e.g., Michael acceptors; Schiff-base formers) improve potency prediction and extend to harder chemistries.^41–43^ For aquatic narcosis, partitioning-anchored physics-based descriptors (e.g., the liposome-water partition coefficient *K*_*lipw*_, and speciation-corrected liposome-water distribution ratio *D*_*lipw* ^(*pH*)^_) provide a mode of action (MoA)-coherent baseline and help flag excess or specific toxicity, including for ionizable compounds.^44,45^ In addition, a computational framework based on continuum-solvated density functional theory (DFT) calculations has been proposed for the screening and design of safer pesticides.^46^ In this approach, physicochemical and electronic descriptors were derived to evaluate both degradability and hazard. Safety thresholds were defined by the octanol–water partition coefficient (log *D*_o/w_) at pH 7.4, a distribution coefficient that reflects compound partitioning between water and organic phases and serves as an indicator for bioavailability and persistence, and by the highest-lowest unoccupied molecular orbital (HOMO–LUMO) gap (Δ*E*), an electronic parameter related to molecular stability and reactivity that provides insight into the susceptibility of pesticides to indirect photodegradation. When applied to approximately 700 pesticides registered by the U.S. Environmental Protection Agency, only 52 compounds satisfied these criteria, demonstrating the potential of this *in silico* strategy to guide the design of safer pesticides.^46^

Despite the broad usability of QSAR models in impact assessment, data scarcity and imbalance in available training data sets remain significant challenges, especially for advanced materials. This issue is fundamental, as the reliability of prediction models is strongly constrained by the amount and quality of empirical data. While synthetic data generation has been explored as a workaround, it introduces uncharacterized uncertainties and cannot substitute for empirical or epidemiological evidence. Recent advances in Artificial intelligence (AI)-driven automated data extraction offer a more sustainable solution to expand high-quality data sets and address the problem of data scarcity. Ha et al. (2025) demonstrated that large language models (LLMs) can systematically extract physicochemical and toxicological information on nanomaterials from the scientific literature.^47^ In parallel, automated machine learning (AutoML) workflows offer complementary advantages. Although they do not resolve data scarcity, AutoML approaches can automate and optimize the modeling process.^48^ For instance, Xiao and Trinh et al., developed nanotoxicity prediction models using three AutoML platforms (Vertex AI, Azure, and Dataiku) based on publicly available data sets for oxide and metal ENMs.^49^ This study showed that AutoML-based models outperformed conventional ML approaches in predictive accuracy and reliability. Nonetheless, AutoML remains dependent on the quality and representativeness of input data, and its black-box nature may limit mechanistic insight and regulatory acceptance. Importantly, QSAR-based prediction, and more in general ML-based prediction should be systematically compared with empirical and epidemiological evidence to ensure relevance and reliability in real-world assessment.^50,51^

Furthermore, QSAR, while valuable, must be applied cautiously due to activity cliffs, whereby small molecular changes cause large biological effects.^52,53^ Another QSAR relevant challenge is related to the interpretation of their predictions, especially in cases where multiple models are used. To derive an overall and reliable prediction, a certain level of expertise is needed. For instance, within the European Partnership for the Assessment of Risks from Chemicals (PARC), a guidebook has been developed for interpreting the QSAR predictions from the VEGA software and drawing an overall conclusion.^54^ However, structural predictions alone do not capture the quantitative relationship between exposure and effect, which can be addressed by dose–response models.

## Dose–Response Models

Dose–response models are essential for human and environmental health impact assessment, providing a framework to describe cause-effect relationships and to extrapolate PoD, which represent doses at which minimal or no adverse effects occur. Traditionally, PoDs were determined using the no-observed-adverse effect level (NOAEL), the highest exposure level at which no adverse effects are observed in an exposed population. However, NOAEL presents limitations, including study design constraints such as sample size, dose spacing, and dose selection, as well as variability across studies and failure to account for response heterogeneity.^55^ To overcome these limitations, the benchmark dose (BMD) modeling methodology was developed and is now considered the state-of-the-science approach for PoD estimation.^56^ BMD modeling involves fitting multiple dose–response models to experimental data, selecting the best-fitting model, typically based on the Akaike information criterion,^57^ to derive the BMD. The BMD represents the dose that elicits a predefined biological response, known as the benchmark response (BMR), commonly set at a 5% or 10% change relative to control, though its level can be adjusted based on biological considerations. Unlike NOAEL, BMD enables dose–response interpolation beyond tested doses and accounts for response variability. Confidence interval derivation further allows quantification of model uncertainty.^58^ The latest European Food Safety Agency (EFSA) guidance document on the use of BMD modeling in the context of risk assessment suggests defining a reference point based on the lowest upper bound of BMD (i.e., BMDL) or by considering the set of intervals and relevance of distinct end points.^59^ Additionally, major health-related agencies, including the National Institute for Occupational Safety and Health (NIOSH), the World Health Organization (WHO) and EFSA advocate for model averaging over individual model selection in BMD derivation to improve robustness and regulatory acceptance.^60^ Nevertheless, there are also limitations related to the BMD application in SSbD. These types of models require high-quality multidose data sets which are usually not available in early innovation of designing and developing a chemical or material, where data uncertainty is high and availability of experimental data is limited. Moreover, the outputs of dose–response models are currently not well aligned with the indicators for hazard characterization established by the SSbD framework.

Considering that dose–response models characterize the relationship between external exposure and observed effects, toxicokinetic models can complement this by describing the absorption, distribution, metabolism, and excretion (ADME) processes that determine internal dose dynamics.

## Toxicokinetic Modeling

Toxicokinetic (TK) models are fundamental tools for human and environmental impact assessment. TK models employ differential equations to describe how ADME processes of chemicals occur in different organisms as a function of time and dose, assessing chemical fate after exposure. Classical TK models use simple compartment representations for organs and tissues but lack detailed physiological or anatomical characteristics, with parameters typically estimated through *in vivo* data fitting. In contrast, PBK models incorporate these physiological characteristics, representing tissue compartments as interconnected systems via the bloodstream.^61^ PBK models capture mechanistic interactions following chemical exposure and translate external exposure into internal burden across multiple routes, enabling risk assessment at the tissue level by comparing target site concentrations to internal PoDs. Their mechanistic nature allows extrapolation across species, demographics (e.g., age, sex), exposure routes, exposure durations, exposure levels or concentrations, and physiological states, including disease conditions. Additionally, they assess the TK of chemical mixtures, like drug–drug interactions in pharmacology.^62,63^ PBK models also incorporate physicochemical properties of the compound under study, as well as biochemical parameters that describe the interaction between the compound and the biological system.^64^ Parameter uncertainty and variability can be propagated to the predicted concentrations through the structural equations, allowing attribution of observed TK variability at the population level to the variability of specific parameters.^65^ Therefore, by accounting for this variability, PBK models support probabilistic risk assessment, allowing for a more comprehensive evaluation of potential risks across different populations and exposure conditions.^66,67^ There are, however, some identified challenges to PBK modeling, including the need for extensive data to develop a model, the frequent lack of data for substances such as novel chemicals or ENMs and the limited availability of concentration data for validating the model.

PBK modeling also facilitates the translation of *in vitro* doses to real-life exposure as part of quantitative *in vitro-in vivo* extrapolation (QIVIVE). Two main approaches are used to extrapolate *in vitro* findings to *in vivo* settings. In the first approach, *in vitro* data are used to directly estimate a PoD, expressed as a concentration. PBK models are then applied through reverse dosimetry to estimate the external exposure level and duration needed to achieve this concentration in the relevant tissue..^68–70^ The second approach involves first translating each *in vitro* concentration from the dose–response curve into an external dose. Then, based on the external dose–response data, the external PoD can be determined.^71–73^ The main outcome of the extrapolation process is usually a human equivalent dose (HED). Simpler toxicokinetic models have also been used for QIVIVE. For example, single-compartment models can simulate the steady-state concentration in the blood for a given exposure. By multiplying the ratio of external exposure to steady-state blood concentration by the *in vitro* outcome, the HED can be determined.^74–77^ The QIVIVE framework serves as a powerful resource for screening novel chemicals and providing insights into their safety.

An equally critical aspect of QIVIVE is *in vitro* TK. For a given chemical and assay type, the measured end point is linked to a specific target site related to the chemical’s mode of action, defining the biologically effective dose (BED).^78^ While the nominal concentration may suffice when most of the chemical reaches the target site without significant binding or loss, processes such as abiotic degradation, evaporation, binding to plastics, binding to proteins and other medium components, or partitioning to off-target cell components often reduce bioavailability.^79–82^ In these cases, metrics like the free chemical concentration in the medium and the cellular concentration (bound and free chemical in the cell) are more appropriate.^83^ In addition to direct measurement of these metrics *in vitro*, *in silico* methods can be employed to estimate them. Various approaches have been proposed in the literature, including equilibrium partitioning models and dynamic models expressing temporal changes through solving a system of ordinary differential equations (ODE). For a comprehensive review on *in vitro* kinetics models, the reader is referred to.^84^ An additional consideration in the QIVIVE framework is the choice of dose descriptor, which can be represented either by the maximum concentration (Cmax) or the area under the curve (AUC), with the latter accounting for the impact of exposure duration.^70^ Given TK models describe the processes governing internal dose and chemical distribution within the body, TGx-based approaches, such as transcriptomics, can complement this by revealing how these internal concentrations translate into molecular responses.

## Toxicogenomic Based Models

TGx leverages omics technologies (i.e., transcriptomics, proteomics and metabolomics) to examine the effects of chemicals and pharmaceuticals on human and environmental health. The vast amount of TGx data available on public repositories represents an invaluable source of potential new knowledge. In this context, TGx data curation is a critical preliminary step, essential for ensuring the quality and reliability of any subsequent investigation.^85^ Well-established pipelines for the preprocessing and analysis of TGx data are widely available, enabling robust and reproducible analyses.^86,87^ The combination of multiple analytical models and pipelines facilitates the resolution of complex biological questions.^86,88^ Advances in BMD modeling of transcriptomic data have led to the development of multiple tools, recognized as promising approaches for toxicity testing.^89–94^ Extrapolating toxicogenomic data to humans is still challenging, though, as cellular responses can differ depending on the model used (e.g., *in vitro* vs *in vivo*, cell types).

TGx approaches are particularly valuable for identifying early molecular events (also called MIEs) and for elucidating the mechanistic basis of chemical exposure.^86,87,95^ For instance, using such an approach, Morikka et al. identified a subset of immune genes that exhibited a sustained dose-dependent and differential expression response to profibrotic challenge in THP-1 macrophages exposed to bleomycin.^96^ Del Giudice et al. identified a common molecular response to ENM exposure regulated by the zinc finger (C_2_H_2_-ZNF) transcription factor family through meta-analysis.^9^ Dose-dependent analysis revealed that C_2_H_2_-ZNFs regulate 55.3% of dose-dependent genes across diverse conditions. Similarly, Ha et al.,^97^ and Perumalsamy et al.,^98^ applied single-cell RNA sequencing (sc-RNaseq) to investigate immunological responses to silver nanoparticles (AgNPs), revealing upregulation of metallothionein (MT) genes associated with metal ion homeostasis, while pro-inflammatory genes (IL-1b, CCL3) were downregulated, suggesting an absence of strong inflammatory responses. These findings highlight the advantage of single-cell transcriptomics in understanding NP-induced immune modulation.

Transcriptomic-based PoDs (tPoDs) derived through BMD modeling are considered a protective (precautionary) method for evaluating the toxicological effects of chemical stressors.^99^ Deriving tPoDs requires careful evaluation of the experimental design, considering sample size, management of confounding variables, and strategies to mitigate batch effects.^95^ Key technical considerations show that tPoD values are more dependent on dose range and spacing than on the number of replicates, with broader dose spacing reducing uncertainty in results.^100^ BMD derived tPoDs can be generated from short-term exposure studies, providing results comparable to those obtained from long-term tests of apical end points.^101–103^ The consistency of this trend is evident regardless of exposure parameters and also across diverse experimental conditions, such as chemical features, species, sex, and technology platforms.^104^ This combination of efficiency and protective capacity makes tPoDs a valuable tool for risk assessment and for establishing reference doses with greater conservatism and reliability.

Furthermore, tPoDs can be linked to specific biological pathways or key events, facilitating potential biomarker identification.^9–11^ All these could contribute to hazard identification and characterization (PoD derivation) in the SSbD context. However, TGx models may not be suitable for the early stages of innovation, where data is scarce and the chemical space is being explored, since they require data (i.e., physicochemical or biological data) that are typically generated later in the innovation process. While SSbD explicitly allows the use of nonregulatory-accepted NAMs as a premarket approach, TGx-based methods are not yet fully standardized and aligned with regulatory outcomes, and do not yet provide limit values that support consistent decision-making within the currently established SSbD framework. Though TGx-based models provide detailed mechanistic insights into biological responses, read-across and grouping approaches can offer a pragmatic solution for hazard assessment when experimental or omics data are scarce, leveraging structural and functional similarities among substances.

## Read Across and Grouping

Read-across involves inferring information on a target chemical based on similarities to well-characterized source chemicals. This approach often employs grouping strategies to establish meaningful relationships based on physicochemical properties or theoretical descriptors. Nonetheless, read-across also presents constraints with regard to data availability as suitable, high-quality reference substances are often lacking, especially for novel, complex, or UVCB materials. This restricts its applicability in the SSbD assessment process, especially during earlier innovation. Additionally, another existing read-across limitation is related to lack of standardization methods and tools for read-across, but also lack of harmonized criteria for analogue selection, data integration and uncertainty assessment.

Although many practical read-across cases begin with visual structural similarity, higher-confidence practice anchors similarity to AOP KEs using mechanistic metrics; e.g., QM electrophile reactivity within sensitization domains and partitioning-based metrics for narcosis/specific-toxicity separation.^41,43–45^ This approach aligns with peer-reviewed read-across practice frameworks emphasizing explicit hypothesis, mechanistic coherence, and structured uncertainty characterization.^46,105,106^

For ENMs, complexity increases due to their unique properties. Grouping by biological activity, such as toxicity end points, offers useful insights but lacks detail on ENM-specific characteristics and does not fully capture the range of responses.^107^ Omics data can also be used to group chemicals and materials, enhancing insights into biological responses and holistic chemical-biological interactions.^11,108^ Combining biomarkers selected from multiomics data with toxicity end points incorporates genes that are key to the ENM induced mode (or mechanism) of action (MOA).^109^ A recent study used an AOP-based framework combined with TGx to group ENMs by their molecular MOA and ENM potency. The authors identified that chemical composition and size-dependent properties of ENMs, plus biological descriptors, influenced the grouping.^110^ Moreover, advances in ML and molecular docking have significantly enhanced the capabilities of read-across approaches. One recent development is the robust model for predicting novel peroxisome proliferator-activated receptor delta (PPARδ) agonists.^111^ This model uses clustering algorithms to define the AD and to classify activity, a notable improvement over traditional methods.

## Digital Twin Approaches: *In Silico* Chemicals, Materials and Biological Models

While read-across and grouping approaches leverage structural and mechanistic similarities to fill data gaps, emerging digital twin technologies take this concept further by creating dynamic, virtual model of a physical object, system, or process used to simulate and predict its behavior throughout its life cycle.^112^ Unlike simulations, digital twins use digital models, either physics-based or data-driven, to run and analyze various simulations and processes. In the fields of materials and chemicals, digital twins can be applied in their (re)­design phase for (early) hazard and exposure assessment,^112^ digital LCA estimation and circularity,^113^ and process optimization for manufacturing purposes.^114^ Digital twins enable virtual prototyping of new chemical products and processes, allowing computational optimization of product formulations, reaction conditions, and production techniques before physical testing and implementation. Building on this concept for ENMs, tools such as NanoConstruct,^115^ NanoTube Construct,^116^ and ASCOT^117^ exemplify an advanced approach to the creation of virtual ENMs by precise digital construction, energy minimization, and descriptor calculation of nanoparticles, nanotubes, and nanosheets, respectively. These platforms can act as the mathematical framework basis for the development of a digital twin for the (re)­design of materials (Figure S1).^118^ Digital twins are also increasingly applied for *in silico* design of molecules. A key example is the *de novo* drug design, a computational approach that generates novel molecular structures from atomic building blocks with no a priori relationships and without a starting template.^119,120^

Moreover, digital twin representations of cells, tissues, organs, and even human models are emerging, using computational models (e.g., ODE) to simulate how pathway dynamics influence whole cell functions.^121,122^ By integrating gene expression patterns with pathway knowledge, these systems identify networks behind cell functions like growth, contractility, and secretion. These systems, that can be seen as part of digital twins of organs (Figure S2),^122^ can simulate results of *in vitro* or *in vivo* experiments, such as drug delivery or cell-substance interactions.^121^ Furthermore, the emergence of generative AI further enhances the digital twin-based preclinical drug discovery, as well as the early assessment of drug efficacy through the simulation of clinical patient trajectories in the form of phase 1 clinical trials.^121^ In addition, physics-based distribution/transport metrics connect hazard with TK and fate: conductor-like screening models COSMOmic/COSMO-RS compute membrane–water partitioning for PBK and distribution, while structure–permeation relations quantify skin permeability to support absorption modeling and design-space exploration.^123,124^

Recent work on toxicology has demonstrated how digital twin approaches that integrate QSARs with physics-based simulations, using mixed quantum and classical simulations, such as QM, Molecular Mechanics (MM), and Monte Carlo (MC), can substantially overcome the limitations of traditional descriptor-based QSARs.^125^ The Computer-Aided Discovery and REdesign (CADRE) model, which integrates QM/MM/MC with QSAR analysis, predicted skin sensitization potency of active pharmaceuticals ingredients (APIs) and intermediates, achieving 95% accuracy in distinguishing sensitizers from nonsensitizers and 79% concordance with experimental potency categories in external validation on 345 compounds. In this framework, the classical MC component samples solute–solvent configurations and interaction energetics (Coulombic and van der Waals) across lipid bilayer mimetics, while accounting for conformational flexibility and ionization states, thereby enabling accurate calculation of permeability coefficients. These descriptors are then coupled with quantum-derived reactivity indices (e.g., frontier orbital energies, Fukui functions) and combined in statistical models to assess sensitization potency. This performance exceeds that of classical QSARs built solely on generic descriptors such as solubility (log*S*) or molecular weight, which often fail under external validation due to training-set bias. By mechanistically modeling biological events such as dermal permeability, metabolic activation, and protein haptenation, CADRE provides a principle-driven foundation that is now widely used by the pharmaceutical and personal care industries in the absence of *in vivo* and *in vitro* data. This type of mechanistic digital twin approach not only supports occupational safety assessments but also extends to environmental fate domains, guiding regulatory decision-making and the design of safer, more sustainable chemicals.

Digital twins enable dynamic, system-level simulations for process optimization, while Life Cycle Assessment (LCA) extends the analysis to encompass the entire life cycle of a product, providing a comprehensive view of the environmental impacts. Combined they can contribute to the development of automated impact assessments.^126^

## Life Cycle Assessment (LCA)

LCA is a quantitative way to evaluate potential environmental impacts associated with a product system throughout its entire life cycle.^127,128^ It consists of four interconnected phases: goal and scope definition; inventory analysis; impact assessment; and interpretation. While a valuable tool, the application of LCA to novel materials exposes critical limitations, primarily related to data availability and modeling characterization. The first phase, the goal and scope definition establishes the purpose of the study, functional unit, reference flow, and system boundaries.^129^ Following this, in the life cycle inventory (LCI) analysis phase, relevant inputs and outputs across the life cycle of the product or process are identified, compiled, and quantified,^130^ using databases like Ecoinvent^131^ or direct data collection. For novel and advanced materials inventory data are often missing due to confidentiality, proprietary claims, or weak reporting.^132–134^ Analysts therefore rely on proxies, assumptions, or surrogates, introducing substantial uncertainty into the LCI.

The impact assessment (i.e., LCIA) phase evaluates the potential impacts of the product or process using the inventory data.^135^ While various LCIA methods exist, they all rely on classifying emissions into impact categories based on their environmental effects.^136^ This classification requires Characterization Factors (CFs), science-based multipliers used to convert the mass of a substance emitted (e.g., 1 kg) into a common measure of potential environmental impact (e.g., a toxicity score). The lack of reliable, substance-specific CFs is the primary barrier to robustly assessing advanced substances such as PFAS and ENMs. The USEtox model, recommended by the European Commission’s Product Environmental Footprint (PEF) framework to evaluate the toxicity of chemical emissions on human health and ecosystems,^137,138^ was originally developed to assess the impact of organic and inorganic chemicals.^139^ Consequently, its fundamental assumptions break down when applied to such substances.^140^ For instance, the extreme persistence, amphiphilicity, and protein binding properties of PFAS break the foundational logic of the partitioning-based fate model, which relies on the octanol–water partitioning coefficient.^141,142^ Similarly, nanomaterials cannot be adequately represented by chemical composition alone, and require consideration of size, shape, coating, and surface transformations.^143,144^ Furthermore, the derivation of CFs for advanced materials typically relies on scarce and inconsistent toxicity data, as well as on extrapolation factors originally developed for conventional organic chemicals^145–147^ To address some of these limitations, the SimpleBox environmental fate model has been modified to create SimpleBox4Nano (SB4N), which includes rate constants to describe the colloidal processes characteristic of ENMs.^148^ Recent work has integrated USEtox and SB4N models to compute fate factors (FF)^149^ and effect factors (EFs) from *in vitro* data for ENMs.^145^ These efforts are a significant step toward USEtox4Nano which would enable the calculation of the CFs required for LCIA.^143,148^

Finally, in the interpretation phase, the LCI and/or LCIA results are analyzed in relation to goal and scope of the study.^150^ Techniques such as contribution analysis (to identify key contributors to impacts), perturbation analysis (to assess the influence of data variations), or uncertainty analysis (to evaluate result reliability) are employed during this phase.^151^

There is a broad range of software tools to conduct LCA, including proprietary and open source. The most popular tools include commercial software like SimaPro,^152^ and open-source OpenLCA,^153^ and Brigthway.^154,155^

While Life Cycle Assessment (LCA) primarily addresses environmental impacts, Social-LCA extends this framework to incorporate social and socioeconomic dimensions, enabling a more holistic sustainability evaluation.

## Social LCA (S-LCA)

The scope of S-LCAs is the entire life cycle of the product or service assessed. The areas of protection in S-LCA concern human well-being, health, and dignity.^156^ These are operationalized into either thematic or stakeholder-focused impact categories which, depending on the framework, are further divided into impact subcategories and associated indicators (e.g., psia, 2022;^157^ UNEP, 2020;^158^ WBCSD, 2016 ^159^). Compared to the largely standardized environmental LCA, S-LCA remains in the early stages of development,^160^ with a considerable variety of approaches taken.^161–163^ For example, while S-LCA typically follows the phases of LCA (goal and scope definition; inventory analysis; impact assessment; and interpretation), the impact assessment in particular is performed in different ways from study to study, including the use of different indicators, quantification approaches, and ways of aggregating the results.

Multiple commercial databases have been developed for S-LCA (e.g., SHDB, PSILCA, Soca). They are employed with OpenLCA (or other) LCA software to model the inventory and analyze the social performance and risks associated with a product or service. At present, the most feasible way of integrating S-LCA into the SSbD framework is based on a reference scale or type I approach in which the social impacts of a product or service are compared with reference values (which are determined, for example, based on objective performance levels or sectoral averages) to determine the performance level of the analyzed product or service. While the reference scale approach does not enable causally assessing the degree of a social impact caused by a production activity, it allows the holistic assessment of the social performance or risk level of a production system across multiple impact (sub)­categories. This approach aligns, in principle, with SSbD in assessing whether a product meets a required or acceptable performance level.^1,2^

The application of S-LCA comes with a few important limitations. First, as with LCA, S-LCA is limited by the availability of life cycle inventory data, which often results from the lack of access to information held by companies. In addition, the quality of S-LCA results often require the collection of site-specific data on the assessed socioeconomic indicators, which may also be withheld by companies even if inventory data is available. Although this issue can be mitigated by utilizing indicator data from S-LCA databases, the additional issue with the databases is that they consist of sectoral (and in some cases global) averages that do not accurately represent the conditions of the analyzed product or service. Finally, as mentioned above, the lack of standardization in S-LCA methods means that there is no consensus on a single approach as a way forward, resulting at present in a situation where there is relatively little convergence in the developments toward a more robust methodological basis. While S-LCA addresses the societal and ethical dimensions of sustainability, exposure and environmental fate models are essential to contextualize these assessments by estimating the distribution, persistence, and potential human and ecological exposure to chemicals in real-world environments.

## Exposure and Environmental Fate Models

Exposure models assess human and environmental exposure to chemicals across three main categories: occupational, consumer, and environmental.^164^ Occupational exposure models estimate the exposure levels of workers to hazardous substances in occupational settings, focusing on workplaces where chemical use is frequent. They assess potential exposure routes such as inhalation, dermal contact, or ingestion during industrial or commercial use of chemicals. ECETOC TRA worker tool,^165^ ART,^166^ and Stoffenmanager^167^ are examples of occupational exposure models, dedicated to chemicals, that have been recommended by ECHA^168^ for use in REACH.^169^ The Stoffenmanager Nano module enables assessment of occupational inhalation risk to ENM.^170^

Consumer exposure models estimate chemical exposure from everyday products based on use patterns and concentrations. ConsExpo Web is used for assessing the consumer exposure to chemicals in various products. ConsExpo Web has also been adapted for ENMs in spray products via ConsExpo nano.^171^ Other models for screening include ECETOC TRA consumer tool^172^ and Consumer Exposure Model.^173^

Environmental exposure models evaluate the exposure of ecosystems to chemicals, focusing on air, water, soil, and sediment and in waste treatment plants.^174^ These models estimate how chemicals behave and degrade in natural environments and predict their impact on wildlife, plants, and overall ecosystem health.^174^ Multimedia fate models use data on environmental emissions, physicochemical and environmental fate properties to estimate the distribution of masses and concentrations in the different environmental media.^175^ They are typically based on fugacity or mass-balanced models, with the Mackey-type fugacity model being one of the most commonly used.^176^

Environmental fate and release models predict chemical behavior and risks postrelease, estimating their spread across environmental compartments. Essential for the SSbD framework, these models support regulatory evaluations, such as identifying PBT or very persistent and very toxic (vPvB) substances. Although qualitative, these types of data could also be generated by QSAR models.^177^ The implementation of multimedia/multicompartment models such as INTEGRA,^178^ SimpleBox for chemicals,^179^ and SimpleBox4nano^148^ determine the Predicted Environmental Concentrations (PECs), supporting impact assessments in various compartments, including microenvironments (e.g., indoors). In relation to ENMs, Nowack (2017) reviewed environmental exposure models for ENMs, including models for material flow analysis and environmental fate models.^180^ The review highlighted the crucial issue of missing validated PEC values arising from the challenges associated with detecting ENMs in the environment and the high uncertainty of input data. The exposure models, regardless of their targeted population, present limited applicability domain concerning complex substances, such as polymers, UVCBs, as well as the different exposure routes (oral, dermal, inhalation), where most occupational exposure models are inhalation-focused.^181^

## Model Integration for Impact Assessment

The previous sections outlined individual models designed to assess distinct aspects of chemical safety and sustainability. While these models provide valuable insights, their isolation often result in a fragmented approach to safety and sustainability. This lack of integration hinders the understanding of interrelated effects and complicates the development of comprehensive risk management strategies.

Here we focus on literature where models have been integrated to perform impact assessment. Specifically, we examine approaches that aim either to get a more reliable prediction of a single end point, such as through consensus integration, such as through consensus integration (Figure 2A), or approaches that can capture interactions between diverse impact categories such as weighted aggregation and pipelines (Figure 2B, 2C).

**Figure 2:**
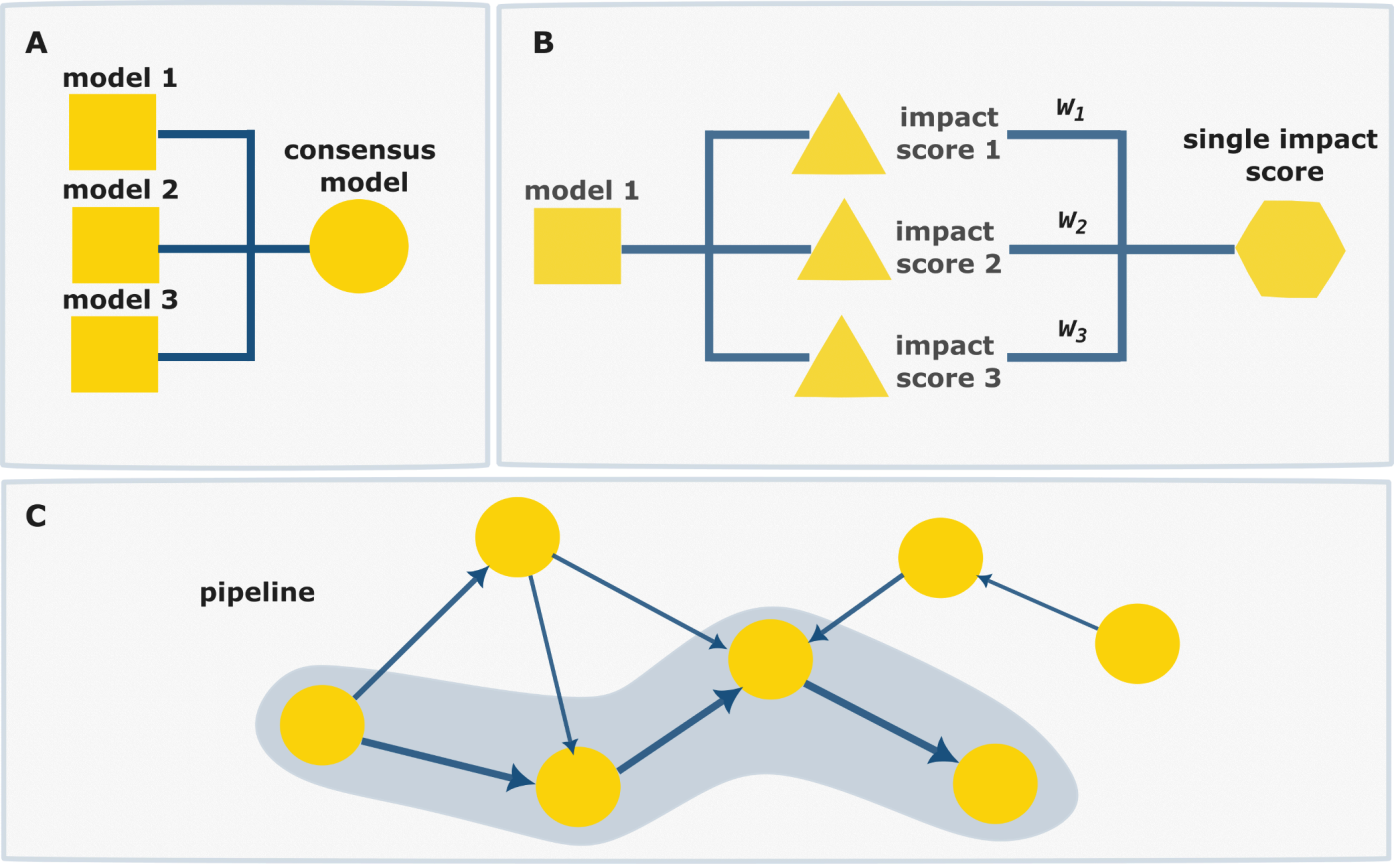
A) A consensus model involves integrating multiple models to predict the same impacts thereby enhancing the reliability of the prediction. B) a consensus score modeling involves integrating multiple scores generated by a model, either within or from different impact categories, into a single score. C) A modeling pipeline connects compatible models through their inputs/outputs allowing an integrated and comprehensive assessment.

This integration gives rise to meta-models, which are composite models made up of individual models. Meta-models enhance computational efficiency by reducing calculation time and power consumption while maintaining accuracy. This integrated approach can be seen as the foundation for a holistic understanding of chemical safety and sustainability, supporting more informed decision-making (Figure 3).

**Figure 3:**
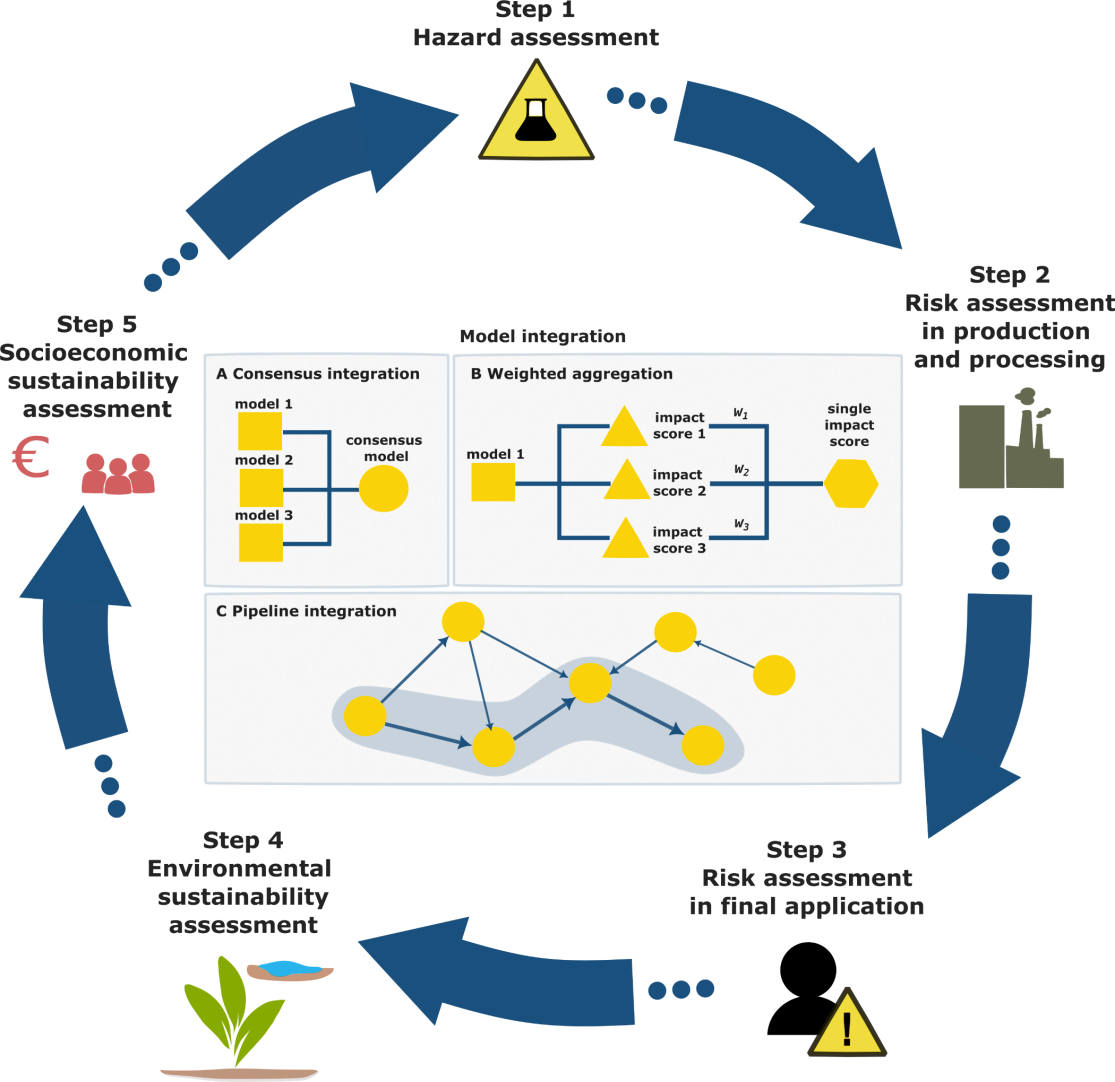
Combining models addressing specific safety and sustainability aspects (through consensus integration, weighted aggregation, and pipelines) can enhance and accelerates the impact assessment of chemicals and materials in the SSbD framework. Figure based on the 2022 EC SSbD framework.

## Consensus Integration Strategies to Predict the Same End Point

Consensus strategies assume that combining several models into a consensus model increases outcome reliability. Multiple consensus or ensemble strategies exist that combine model predictions (Figure 2A).^182^ For example, predictions from QSAR models can be integrated using voting, Pareto ranking, or the Bayes consensus with discrete probability distributions.^183–185^ Voting methods are categorized as hard or soft based on the type of predictions models provide.^186^ In hard voting, the most frequently predicted class is selected. In soft voting, class probabilities are averaged or weighted, and the class with the highest probability is chosen. The Bayesian consensus method updates probabilities using the Bayes rule to determine the final class based on model outputs. Ensemble methods, such as bagging, boosting, stacking, meta models, and a mixture of expert models, combine multiple model predictions to improve overall performance by leveraging the strengths of each. The consensus modeling concept can be applied to predictive models for SSbD. For example, Ji et al. used an ensemble of models, including Random Forest and XGBoost, to predict the chronic developmental toxicity of PFAS in threatened and endangered fishes.^187^ Schieferdecker et al. developed a consensus model for prediction of Oral Acute Rodent Toxicity of multiple chemicals using the global harmonization system (GHS) categories.^188^ Mastrolorito et al., developed a public web platform that is connected to a consensus model for the prediction of developmental toxicity for chemicals.^189^ Moreover, in Varsou et al., multiple ML models and quantitative read-across structure–property relationship (q-RASPR) models were trained independently on a shared data set and integrated into a consensus framework, using simple and weighted averages, to predict the zeta potential of ENMs.^190^ The consensus model outperformed all individual models, resulting in more reliable predictions. Therefore, this approach has been proposed as a valuable method for enhancing reliability and regulatory acceptance of nanoinformatics tools.

While consensus integration strategies combine multiple models to improve predictions for the same end point, weighted aggregation methods address the challenge of integrating multiple impact categories into a single evaluation score. The next section illustrates how this approach is applied in the context of LCA.

## Weighted Aggregations of LCA Results

LCIA methods distinguish between midpoint-level indicators and damage-oriented indicators (Figure S3). Each emission can contribute through multiple impact pathways to one or more of the so-called areas of protection (AoP): human health, ecosystem quality, and resources (Figure S3). Damage indicators provide an overview of aggregated effects at the AoP level, but their use in decision support is limited by additional layers of complexity and uncertainty. When results are aggregated across multiple impact categories to support decision-making, this is typically done at the midpoint level, where indicators are more closely tied to underlying environmental mechanisms and thus less uncertain. Aggregation proceeds in two sequential steps: normalization, followed by weighting (Figure 2B).^138^ Normalization converts characterized impact scores, typically expressed in different units, into a common reference scale. This is achieved by dividing the impact scores of the analyzed system by reference values representing broader systems, such as national, regional, or global emissions and resource use (external normalization) or a baseline alternative (internal normalization). Normalization helps contextualize results and is often a preparatory step for weighting. Weighting assigns relative importance to different impact categories, allowing their aggregation into a single score. This step facilitates interpretation and communication by reflecting the perceived significance of impacts. Weighting factors are inherently value-driven and may derive from policy targets (e.g., distance-to-target weighting), stakeholder preferences, or economic valuation.^191^ While the ISO 14040–14044 series consider normalization and weighting as being optional,^127,192^ the EC’s PEF method mandates these steps to facilitate decision-making.^137,138^

In practice, the PEF method employs external normalization based on global emissions and resource use for 2010.^193^ However, normalized results should be interpreted cautiously, as they do not reflect impact severity or trade-offs between categories. The choice of reference values and inventory completeness can significantly influence outcomes. Since normalization treats impact categories independently, it should supplement, rather than replace, characterized impact scores.

The JRC promotes a hybrid approach to weighting combining evidence-based methods and expert judgment.^194^ This involves a two-step procedure:

1) Weighting impact categories indicators within the end point areas of protection: human health, natural environment, and natural resources to simplify preference quantification.
2) Weighting the three end point categories to reflect broader perspectives and societal values.

The JRC emphasizes that weights should be derived from a combination of public preferences and expert input. Since weighting inherently involves subjective value choices, transparency is paramount. Consistent application of a single weighting set across different product groups is also critical to ensure the comparability of LCA results.

In S-LCA, the integration of results across different indicators is typically done by expressing the value of each indicator in the same unit. The results are aggregated at the impact (sub)­category level to provide an overview of the aggregate risk levels of the analyzed product. It is also possible to apply multicriteria decision analysis methods to achieve noncompensatory aggregation, i.e., assessing the aggregate performance of a product in a way that does not allow poor performance in one indicator or impact category to be compensated by good performance in others.^195^ In causal or type II S-LCA approaches, efforts have also been made to assess the health-related social impacts particularly by expressing them as a single unit at end point, such as Disability-Adjusted Life Years (DALYs) or Quality-Adjusted Life Years (QALYs).^196,197^ The integration of social, environmental, and economic assessments faces challenges due to differing methodologies and maturity levels between the three approaches.^198,199^ As a result, the existing work has focused primarily on the integration of results rather than the interconnections among underlying models, employing results aggregation for decision support.^200^

## Combination of Models into Pipelines

While weighted aggregation combines model outputs at the end point to provide an overall sustainability score, pipeline integration takes a different approach by connecting models in a sequential manner, where the output of one model becomes the input for the next, resulting in a more comprehensive and cohesive impact assessment (Figure 2C).

An established integration of models is employed in risk assessment, where the output from dose–response modeling, often implemented through BMD modeling, is linked to concentrations predicted by TK modeling of external exposure. This modeling pipeline can follow two distinct configurations based on the desired outcome. In the first approach, the model outputs are compared to derive risk-relevant insights related to exposure. In the second approach, the dose–response model output serves as input to the TK model, which is then applied in reverse dosimetry to estimate the corresponding human equivalent dose (HED). Incorporating dose–response relationships into PBK models can provide a biological basis for understanding sources of potential data variability related to differences in exposure routes, types of studies, study design, and species. The integration of PBK within QIVIVE to translate PoDs to HED has been shown to be useful for a quantitative interpretation of *in vitro* model responses,^201^ as well as potential species differences for some interspecies extrapolations.^202^ These integrative approaches provide a biologically relevant framework for translating *in vitro* and *in vivo* data to human contexts.

Advanced computational workflows have been developed around PBK models that support prediction of internal disposition following inhalation exposure. The integration of occupational exposure models, such as the multibox aerosol model, with PBK models enables detailed assessments of ENM biodistribution across human organs after inhalation. By incorporating clearance mechanisms and dynamic concentration–time profiles, these models improve the accuracy of risk assessments for both acute and chronic exposure scenarios.^203^ In particle inhalation situations, dosimetry models like the multiple-path particle dosimetry model are employed to estimate the fraction of particles deposited, which subsequently informs PBK simulations.^204^ Additionally, computational fluid dynamics models provide critical insights into aerosol deposition and gas absorption across lung regions, offering a first-principles perspective that serves as input for simulating *in vivo* biodistribution via PBK modeling.^205,206^

Recent efforts have integrated PBK models and TGx approaches.^202,207,208^ Chen et al. reported a method of integrating dose-dependent omics data into PBK models to estimate reference doses for chemical risk assessment.^202^ Using perfluorooctanesulfonate (PFOS) as a case study, differentially expressed genes (DEGs) were identified from human *in vitro* and mouse *in vivo* TGx data across various exposure durations. The dose–response relationships were analyzed using the Bayesian benchmark dose method to determine PoDs, which were then converted to HEDs via a PBK model. These HEDs were aggregated at the pathway and disease levels and extrapolated to reference doses by applying appropriate uncertainty factors. This integrated approach offers a robust alternative for risk assessment by providing mechanistic insights and considering multiple end points without relying solely on traditional apical toxicity end points.^202^

Silva et al. employed a similar omics and PBK modeling approach to derive a tolerable daily intake TDI for perfluorooctanoic acid (PFOA).^207^ By analyzing *in vitro* hepatic transcriptomics concentration–response data, they identified sensitive molecular pathways affected by PFOA exposure and determined molecular PODs using BMD modeling. These molecular data were extrapolated to *in vivo* doses through reverse dosimetry within a QIVIVE framework using a PBK model.

Moreover, integrating PBK models with the AOP framework can facilitate the linking of external dose to internal doses needed to activate MIEs occurring in a target tissue described in an AOP.^209,210^ This integration can help predict the TK and toxicodynamic (TD) profiles of the chemical specific MoA triggered by the exposure. These approaches are key to improving the regulatory implementation of *in vitro* and *in silico* based NAMs. Building on these developments, a potential future NAM-based classification scheme that combines TK and TD information for hazard classification decisions is currently being designed within the European Partnership for Alternative Approaches to Animal Testing (EPAA) “designathon” for human systemic toxicity.^211^

Models can also be integrated to supplement data sets on new chemicals, or those that are less studied and lack sufficient data for new model development. For instance, PBK models require many species-specific physiological parameters and chemical-specific ADME parameters as input. The collection of these data can be time-consuming, and for new chemicals, generating such a large array of input data is unfeasible. To address this challenge, computational pipelines that integrate QSAR or ML/AI models with PBK models can be employed for predicting the required ADME parameters.^212–216^ Complementing these data-driven approaches, atomistic simulations compute free-energy (PMF) profiles and diffusivities across lipid/membrane media via Molecular Dynamics (MD) simulations, yielding quantitative permeability/bioavailability estimates that feed PBK models.^217^ These physics-based methodologies have been applied to predict the blood–brain barrier permeability^218^ and skin permeability,^219^ providing parameters that can be directly incorporated into PBK models. For a detailed overview of *in silico* models supporting PBK parametrization, readers are referred to Madden et al. (2019).^220^

Finally, within the NanoSolveIT project,^221^ model integration was demonstrated in an initial version of the integrated approach to testing and assessment Integrated Approach to Testing and Assessment (IATA) linking predicted indoor air concentrations of nanomaterials to lung dose and biodistribution.^203^ This was further formalized into the NanoSolveIT IATA as shown schematically in Figure S4, which includes an exposure tier, along with characterization and hazard tiers, each utilizing different models and meta-models. The exposure tier addresses both environmental and human (occupational) exposure, subdividing each into external and internal assessment tools. This allows end-users to simulate ENM emissions and internal disposition over time in various media and species. The accumulated dose can then be compared to established PoD to derive risk-relevant conclusions.

## Model Uncertainty

Having examined both individual modeling approaches and their integration into structured pipelines, it is essential to address a critical cross-cutting aspect: model uncertainty. Regardless of whether models are applied in isolation or as part of complex workflows, uncertainty in chemical impact assessments stems from data variability, model assumptions, and scenario definitions, as models are highly sensitive to the initial assumptions about spatial and temporal scales, exposure pathways, and environmental contexts.^222^ Estimating and recognizing uncertainty is essential for regulatory acceptance of *in silico* chemical safety assessment methods.^223^ In this context, is imperative to stress the importance of data preprocessing and curation. Poorly curated input data, erroneous structures, inconsistent units, mislabeled end points, unresolved duplicates, or missing meta-information can undermine even the strongest modeling architecture. In practice the concept of “garbage in, garbage out” holds: if the input quality is compromised, no model architecture can reliably compensate. Thus, careful manual review, provenance tracking, metadata consistency, outlier detection, and chemical structure standardization must remain core steps. Furthermore, external validation is a powerful means of reducing model uncertainty and is relevant across a wide range of *in silico* approaches. In practice, this means testing the model on independent compounds (or data sets) that were not used in model training and ideally lie within or near the intended chemical space of use. External validation helps reveal overfitting, domain extrapolation errors, or hidden biases. This is consistent with OECD recommendations, which emphasize external validation and clear applicability domains as key principles of model acceptance.^224–226^

Uncertainty in QSAR model predictions arises from variability and biases in experimental data and computational approaches.^227^ Factors such as molecular descriptor selection, measurement errors, and data set inconsistencies further compound variability.^228^ To mitigate these uncertainties, cross-validation ensures predictions are not data set-specific, while bootstrapping, MC simulations, and Bayesian methods quantify reliability and provide probabilistic error bounds.^229^ Additionally, characterizing the AD is fundamental to managing prediction uncertainty, guiding reliable use and endure model credibility.^20,230,231^ Indeed, the AD is defined as the chemical space where QSAR model provides reliable prediction. Common AD estimation strategies include distance-based methods (e.g., leverage, Euclidean distance, k-nearest neighbors), range-based approaches such as principal component analysis, geometric techniques like convex hulls, and probabilistic frameworks employing kernel density or class probability estimation.^232–234^ However, AD determination remains challenging because no universal method exists, thresholds are empirically defined, and most approaches apply global binary criteria that ignore local accuracy variations, leading to optimistic reliability. Recent work by Mora et al. proposes integrating inhomogeneity mapping and error analysis into AD workflows, coupled with rigorous validation, as a promising strategy to improve QSAR prediction reliability and enable targeted model refinement.^232^

Similarly, defining the AD is essential for interpreting TGx data in chemical safety assessments, encompassing the tested chemical, biological interactions, exposure time, and dose. TGx data demonstrate that cellular responses depend on the experimental system.^10,235^ Thus, factors such as the biological model, exposure time, and dose levels influence the outcome, underscoring the importance of defining the range of conditions under which data interpretation remains valid and relevant to real-life exposure scenarios, in toxicogenomics studies.^108^ Notably this is true for both *in vitro* as well as *in vivo* test systems, and the assessment target (the human population or ecosystem), may be highly variable. This variability may be driven by (epi)­genetic background as well as various additional interacting real world stressors.

In dose–response modeling, confidence intervals around the BMD quantify uncertainty, defining lower (BMDL) and upper (BMDU) limits to accommodate experimental variability and regulatory flexibility. The BMDU/BMDL ratio, per EFSA guidance, serves as a metric for uncertainty characterization.^59^ BMD uncertainty is influenced by model selection, sample size, and biological variability, affecting sensitivity and consistency. Bayesian BMD modeling allows to build on prior available best model-fits and enhances uncertainty quantification by integrating experimental data via likelihood functions.^236^

Structural uncertainty, parameter estimation, biological variability, and extrapolation uncertainties significantly affect TK modeling.^237^ Different model structures yield variations in internal disposition and HED estimations under forward and reverse dosimetry.^238,239^ Calibration using *in vivo* data is a common way to parametrize TK models. Parameter uncertainty can be captured using statistical distributions within appropriate statistical learning frameworks.^240^ When population data are available, Bayesian modeling facilitates the estimation of interindividual variability.^241,242^ Physiological and anatomical factors, such as blood flow, organ volumes, and enzyme expression, further contribute to variability.^65,243^ Uncertainty analysis is commonly performed using MC sampling, bootstrap resampling, or Latin hypercube sampling,^244^ with sampling importance resampling (SIR) providing a distribution-free alternative.^245^ Coupling uncertainty and sensitivity analysis ensures a more comprehensive evaluation of model parameters.

The validation of LCA analysis can be seen as a multilayered process of verification designed to ensure the integrity, representativeness, and robustness of the overall model. This process must be transparently documented and justified in the accompanying report and explicitly considered in the interpretation phase. At the data level, quality and representativeness are assessed by rigorously evaluating whether inventory data are technologically, geographically, and temporally appropriate. This often involves performing plausibility and correctness checks, such as establishing mass and energy balances, and comparing data against similar processes, published literature, or the opinions of technical experts to find anomalies and confirm validity. At the modeling level, completeness and consistency checks confirm that system boundaries, allocation procedures, and cutoff criteria are applied transparently and uniformly, and that all environmentally significant flows and processes are included. At the study level, the entire LCA is subjected to an independent critical review in line with ISO 14040/14044 requirements, particularly for studies making comparative assertions intended for public disclosure.

While these verification steps ensure the methodological soundness of an LCA, they cannot fully capture the uncertainty inherent in model assumptions, data limitations, and methodological choices. To address this, uncertainty and sensitivity analyses form an essential complement to verification and are explicitly recommended by ISO 14040–14044.^127,192^ Sensitivity analysis evaluates how results change with model assumptions,^246^ while uncertainty analysis quantifies variability in model outputs. Heijungs highlights key factors that significantly impact LCA outcomes,^247^ which include: the selection of the functional unit and system boundaries; the inclusion of behavioral and market aspects; the choice of databases and the data quality for both LCI and LCIA and strategies for handling missing data; the choice of allocation principles for multifunctional processes; the choice of impact assessment method and the decisions within specific impact categories; and the normalization references and weighting principles. However, guidelines regarding the implementation of sensitivity and uncertainty analysis lack operationalization, and the two concepts are not always clearly delineated, such that these recommendations are not always followed in practice.^248^ Monte Carlo simulation is one of the most applied approaches used to analyze uncertainties in LCA.^249^ It generates probability distributions of LCA results by performing multiple simulations based on predefined parameter uncertainties. This allows the calculation of mean, variance, and confidence intervals, improving uncertainty quantification. Monte Carlo analysis often relies on the uncertainty ranges provided by databases like Ecoinvent, the most widely used source for life cycle inventory data. However, while this approach improves on deterministic modeling, it has significant limitations.^250^ A key issue lies in the ’pedigree matrix’ used in Ecoinvent, which qualitatively evaluates data quality along multiple dimensions. In practice, these qualitative evaluations are converted into quantitative uncertainty factors to define probability distributions, a process that involves subjective assumptions and simplifications. This can introduce bias and distort the true representation of data uncertainty, whereas Monte Carlo simulations assume that input distributions accurately reflect the underlying uncertainty. Finally, uncertainty in characterization factors is rarely considered.^151,251^ Most impact assessment methods do not provide quantitative uncertainty information, and LCA software generally lacks the capability to integrate these uncertainties, except for Brightway.

In S-LCA, similar sources of uncertainty apply. Notably, S-LCA comes with significant uncertainties related to the extent to which indicators measure the areas of concern they are linked with, how well they reflect the end point of human wellbeing given the lack of theoretical foundation, and the subjective and contextual nature of social impacts which complicates the interpretation of results. While the reference scale approach mitigates some uncertainties by using performance or risk levels rather than continuous values, the limitations on data availability and quality also introduce uncertainty into S-LCA. Overall, few studies consider uncertainty assessment as part of S-LCA.^252^

## Uncertainty Propagation in Model Integration

When integrating multiple models, the uncertainty propagation and the AD of the combined framework are pivotal considerations. In single-model assessments, uncertainty quantification methods like MC or bootstrapping are tailored to the model, while in multimodel frameworks, interacting uncertainties further complicate reliability estimation. A key challenge in both consensus and pipeline integration is the wide heterogeneity of models available for impact assessment. These models may yield outputs and uncertainty estimation in diverse formats (e.g., probabilistic, deterministic, statistical, or frequentist, as well as range-based) making it particularly challenging to determine the most appropriate approach for evaluating. One possible strategy could be to first conduct a sensitivity analysis of each individual model and quantify its uncertainty. Once the uncertainties of all models have been characterized, simulations can be employed to systematically vary individual model outputs and estimate how fluctuations impact the final combined output. The AD further complicates integration efforts, as it restricts the contexts in which predictions are valid. In sequential pipelines, the AD is limited by the narrowest domain of the individual models (Figure 4A). For consensus modeling, the AD is defined by the overlap of the AD of the individual models (Figure 4B). Defining and managing ADs and understanding how uncertainties propagate within these boundaries, is essential for ensuring model reliability and accuracy in coupled modeling systems.

**Figure 4:**
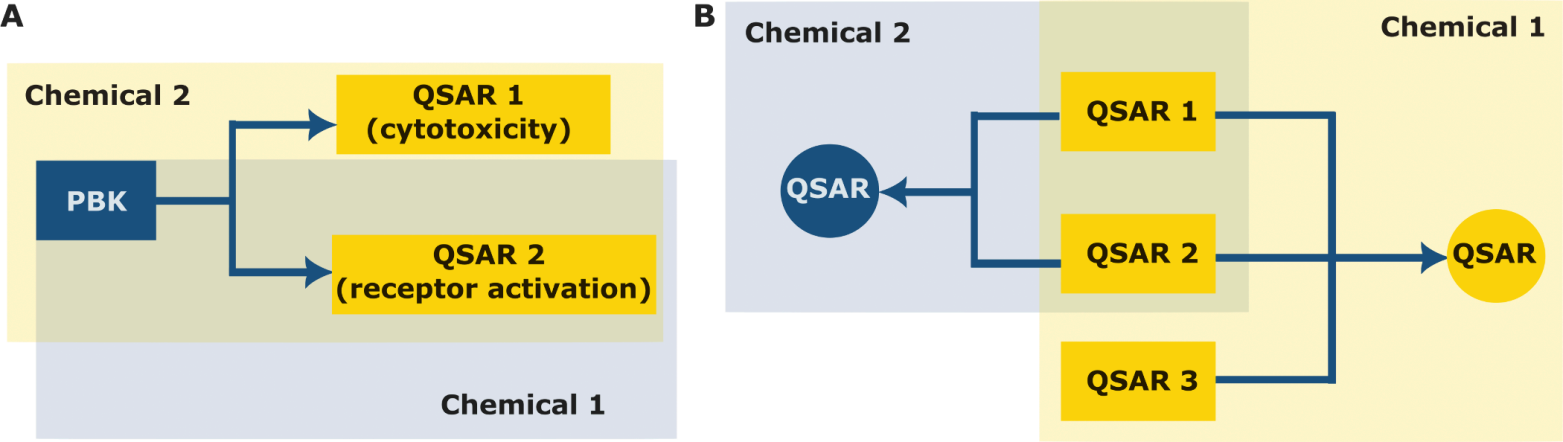
A) Model integration through pipelines. The presented QSAR models function as dose–response models, taking the internal tissue concentration estimated by the PBK model as input to predict either cytotoxicity or the extent of receptor activation. The integration of PBK and QSAR depends on their ADs. In the yellow-colored box, the PBK model is built for chemical 2, and chemical 2 is also in the AD of QSAR 1 and QSAR 2. In the dark blue colored box, the PBK is built for Chemical 1, and this chemical is not in the applicability domain of QSAR 1, therefore QSAR 1 should be excluded from the integration. B) Parallel integration of models. The integration of QSAR models for the same end point (e.g., carcinogenicity), depends on their ADs. In this example, chemical 1 is in the AD of all available models so they can all be integrated. Chemical 2 is only within the AD of models 1 and 2, therefore model 3 should be excluded from the integration.

## Redefining Toxicology: Advancing NAMs and Computational Models for Sustainable Risk Assessment

A fundamental transformation in toxicology is underway, moving from reliance on animal testing toward NAMs and computational models that enable early, mechanism-based decision-making within SSbD frameworks. GHS (Globally Harmonized System) hazard classification has historically relied on animal test data and applies primarily to existing substances with available studies, whereas SSbD is a premarket innovation framework that explicitly enables the early use of NAM evidence alongside other data streams for design-stage decision-making.^3^ By contrast, LCA human and ecotoxicity characterization has traditionally drawn on animal-derived effect data (e.g., USEtox),^139^ with emerging research exploring how *in vitro*/NAM data can be translated via IVIVE/PB­(K) into effect factors, promising but not yet standard practice.^253,254^ However, reliance on animal testing poses well-known constraints that also conflict with broader sustainability goals spanning social, economic, and ecological dimensions and is often impractical for novel substances where no animal dossier exists.^255^ Ethical concerns, high costs, and prolonged testing times hinder the assessment of existing chemicals and delay the evaluation of new, greener alternatives. Additionally, the feasibility of retesting substances to reflect scientific advancements remains limited. Accordingly, for novel chemicals/materials under SSbD, alternative methods (e.g., QSAR/read-across, PBK, TGx, *in vitro*) can provide early evidence even when GHS classifications are not yet available.^3^

Thus, a new toxicology perspective is needed, focusing on molecular and cellular effects, which are testable via *in vitro* and *in silico* methods, so-called NAMs. These molecular and cellular effects function as risk indicators, similar to (epi)­genetic predispositions, influencing an organism’s ability to cope with environmental stressors. Since stressor interactions and genetic diversity cannot be fully tested *in vivo* or *in vitro*, modeling on experimental data is essential. Importantly, animal tests do not necessarily offer better predictive power, but their uncertainties remain less transparent to regulators.^256–261^

From a regulatory perspective, the extrapolation procedures and associated uncertainties for *in vitro* and *in silico* models are conceptually like those used for animal-based data. Both approaches determine the PoD representing minimal effect levels, extrapolate these to target exposures, and incorporate variability modeling. In current regulatory practice, animal-based PoDs rely on deterministic extrapolation factors, whereas *in vitro* PoDs employ PBK modeling. Comparative studies indicate that target variability contributes significantly to overall uncertainty. Increasing transparency regarding uncertainty sources is critical for regulatory decision-making in (eco)­toxicology. To align with this evolving perspective, a new regulatory classification system is required, one that prioritizes an understanding of adversity based on *in vitro* and *in silico* data.

To align regulatory practice with this mechanistic and NAM-centered perspective, a new regulatory classification system is required. Rather than maintaining rigid hazard categories such as carcinogenicity or reproductive toxicity, this new framework would primarily emphasize potency assessments. A pragmatic approach could involve defining likely safe doses along with the most probable adverse effects at higher exposures. This regulatory shift is currently under development through a multistakeholder collaboration led by the European Partnership for Alternative Approaches to Animal Testing (EPAA) and the JRC.^211^

Nonetheless, uncertainties in experimental and computational model outputs create challenges in interpreting results for regulatory or environmental decision-making. A key issue is whether stakeholders accept computational model outputs as substitutes for experimental data. While computational models also carry uncertainty, they are trained on extensive data sets and can estimate values where experiments are infeasible.^262^ Moreover many models have been validated according to the principles of the internationally accepted OECD guidelines for validation of QSAR models.^224^ Building trust in AI/ML models is crucial for their acceptance in risk assessment, LCA, and SSbD. Trust requires proof of relevance, robustness, and reliability, which is best achieved through early collaboration between stakeholders and modelers. When appropriate, risk assessors may benefit from engaging directly with AI/ML tools, provided sufficient training and support are available, rather than relying solely on computational experts. Although this can help build familiarity and confidence, we are not yet at the level where expert mediation is necessary. To support adoption, AI/ML models should be user-friendly, transparent and fully documented (e.g., via QMRF and/or MODA templates), and widely accessible, ideally through platforms that lower technical barriers. Full implementation also requires acceptance from broader stakeholders (e.g., risk managers, industry), regardless of positive or negative outcomes. Finally, transparent reporting of output uncertainty is essential for accurate risk communication and decision-making. A key aspect of ensuring trust in AI/ML models is adherence to FAIR (Findability, Accessibility, Interoperability, and Reusability) principles, which promote transparency, reproducibility, and interoperability in predictive modeling. However, ensuring FAIRness extends beyond data to include software, as predictive models depend on computational frameworks that must be reusable and interoperable across regulatory applications. The FAIR for Research Software (FAIR4RS) framework addresses these challenges by emphasizing software-specific aspects such as licensing, dependencies, versioning, and containerization, ensuring that models remain traceable and executable across different SSbD frameworks.^263^ The European Commission reinforces this need by promoting FAIR principles for metadata, algorithms, and models to enable transparent and interoperable assessment methodologies.^264,265^

Despite its potential, full FAIRification of software remains aspirational, leading to ongoing discussions on the role of community-driven solutions in enhancing FAIRness across regulatory ecosystems.^266,267^ Tools have been developed to assess FAIR compliance,^266,268^ yet challenges persist, including poor metadata quality, inconsistent terminology, and the need for improved model-sharing platforms. Cronin et al. propose FAIR guidelines tailored for *in silico* predictive models, identifying key areas for improvement in metadata standardization and accessibility.^269^ Addressing these gaps will be critical to fostering trust in AI/ML models and ensuring their adoption in regulatory risk assessment.

## Discussions and Perspective

The rapid emergence of new chemicals and materials has intensified the need for robust approaches to evaluate their safety and sustainability. Although the integrated impact assessment across multiple dimensions is required to fully implement SSbD, these evaluations are still frequently performed by different expert communities in a fragmented fashion. This results in the assessment of isolated end points with no complete understanding of how these effects interconnect across biological scales and impact categories. Such fragmentation limits the simultaneous optimization of function, cost, safety, and sustainability.

This review shows that a broad toolbox of predictive models already exists (such as QSARs, TGx, PBK models, exposure models, and LCIA), while a variety of integration strategies (such as consensus modeling, weighted aggregation, and pipelines) have emerged in the literature and can be applied to support comprehensive impact assessment.

In this context, the INSIGHT EU project provides a concrete illustrative case of a unified framework that embeds the fragmented assessment and models.^4^ Grounded in One Health and multiscale modeling, INSIGHT proposes Impact Outcome Pathways (IOPs), an extension of AOP framework across multiple relevant dimensions, to align heterogeneous data and models along causally linked KEs spanning molecular, cellular/tissue, individual, population/ecosystem and socioeconomic levels (Figure 5). This approach reflects the complexity of chemical exposure responses in a holistic manner, in which interactions between substances and biological systems occur across multiple spatial and temporal scales, from molecular and tissue processes to population, ecosystem, and societal outcomes.^8,270^ The IOP thus serves as a multiscale model that operationalizes this integration: it provides (i) standardized KE identifiers and relationships for mapping outputs from diverse models; (ii) a unified framework to integrate evidence strength and uncertainty at each KE/KER; and (iii) clear “interfaces” for passing information between multiple scales.

**Figure 5:**
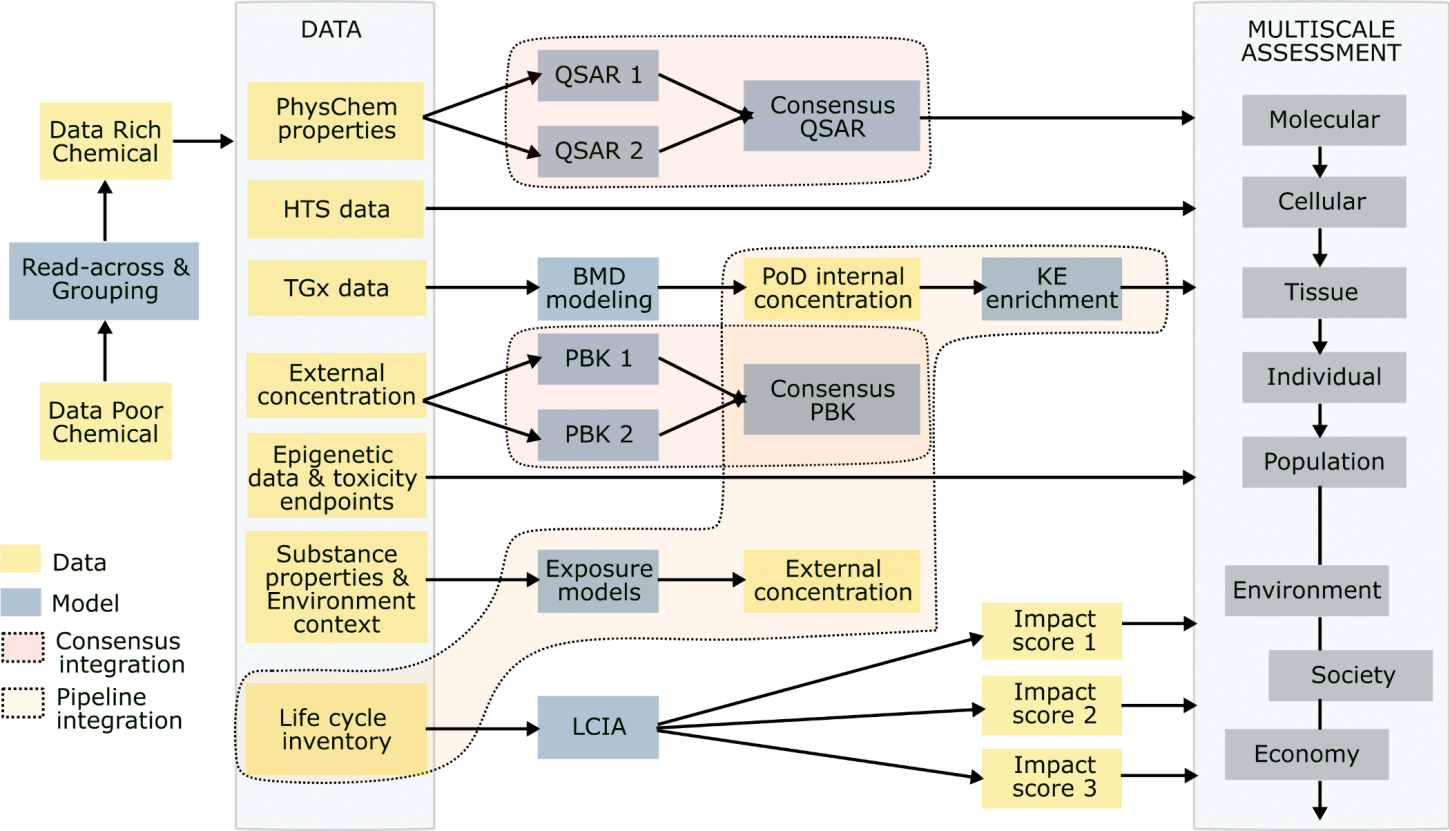
Schematic representation of a multiscale impact assessment modeling application. Yellow boxes indicate data, while blue boxes indicate models. Black boxes indicate different levels of organization of chemical impact in the multiscale assessment framework. Dashed arrows show connections between data, models and impacts. If the chemical under study is a data poor compound, a data rich substitute can be found through read-across and grouping approaches. For data rich substances, different data types will be mapped to the multiscale assessment model by using different types of models. When multiple models to predict the same end point are present, they can be grouped into a consensus model (e.g., QSAR). Moreover, the figure also shows an example of a pipeline that would combine LCA, Exposure models, PBK and TGx modeling. The idea is that LCI data and exposure models can be used to get an estimate of external concentration of chemicals (e.g., leaking chemicals during the production) in the environment. These can be used in input of PBK models to predict internal concentration in tissues that can be then compared with PoDs predicted from TGx data that can then be mapped to KEs and AOPs of the AOP framework to identify possible adverse outcomes triggered by that exposure.

Within this context, each KE can be informed by modeling approaches tailored to the scale of interest. At the atomic and molecular level, molecular dynamics and docking simulations elucidate interactions between chemicals and biomolecules. These can be complemented by QSAR models, which link chemical structure to biological activity, and toxicogenomic approaches, which capture molecular responses via omics technologies. Moreover, High Throughput Screening (HTS) data provide empirical evidence of compound bioactivity across different biological targets that can be mapped to the early KEs of the health impact related portion of the IOP. In parallel, epigenetic data can provide insights into KEs that include regulatory alterations that can mediate persistent phenotypic outcomes. Furthermore, epigenetic data can directly support mechanistic evidence of function as modulating factors influencing the magnitude, duration or persistence of downstream KEs. Tissue- and organ-level responses are simulated through PBK models. Finally, societal, economic and planetary scale consequences are quantified using LCA tools and socioeconomic impact models. By making these linkages explicit, the IOPs can enable cross-model concordance checks (e.g., TGx KE signatures vs PBK-derived internal doses) and make uncertainty propagation and applicability-domain declarations visible at the point of decision.

This approach also facilitates the implementation of integration strategies, such as those reviewed in this paper (Figure 5). Consensus modeling (e.g., consensus QSAR or PBK), combining multiple models addressing the same end point, improves prediction reliability and reduces model-specific biases. Weighted aggregation strategies can be used to combine the results of different impact score estimation into an integrated score. Finally, pipeline-based integration, that connects models across different domains, enables the propagation of information from one scale to another.

For example, LCI data could be used in combination with exposure models to get an estimation of the accumulation of chemicals present in an environmental compartment by a certain production process. These predicted concentrations can be used as inputs to PBK models to calculate the internal concentration of such chemicals under the exposure scenario after a certain duration of exposure. As previously demonstrated by Silva et al.,^207^ and Chen et al.,^202^ PBK and omics-based BMD modeling outputs can be integrated to support risk assessment. Similarly, concentrations in specific organs predicted by PBK models can be compared with the internal concentration of specific KEs inferred from *in vitro* data, such as transcriptomics data (e.g., pathway-level BMD/BMDL). This comparison enables the identification of portions of the IOP network activated by the exposure scenario and clarifies the interconnections between estimated effects. In this way, the IOP scaffold operationalizes pipeline integration: it specifies where each model plugs in, what evidence is transferred, and how concordance and uncertainty are evaluated along the chain.

Despite these advances, achieving a fully operational model integration framework for impact assessment remains challenging. It requires sustained efforts in standardization, harmonization, and open data sharing. The development of automated tools for assessing FAIR compliance, along with enhanced interoperability through harmonized metadata and ontologies, will be essential for seamless data exchange and for fostering trust and acceptance by regulatory authorities. Moreover, mapping heterogeneous NAM outputs to KEs across different biological contexts, exposure routes, life stages and costressors remains challenging and can introduce ambiguity. In parallel, methods for uncertainty propagation, especially in complex, multimodel cascades, are still evolving and will require further refinement to ensure robustness, reproducibility, and interpretability in real-world applications.

In conclusion, this review emphasizes the need for integrated modeling strategies to address multiple dimensions of chemical impact, which in turn can facilitate the implementation of the SSbD framework. While a broad set of predictive models exists, their independent application limits the ability to address complex interactions among safety, environmental, and socioeconomic dimensions. We examined three integration strategies, namely consensus modeling, weighted aggregation, and pipeline integration, and discussed how uncertainty and applicability domains evolve within these frameworks. Addressing these challenges and leveraging initiatives like the EU project INSIGHT will be essential to build interoperable, transparent, and uncertainty-aware modeling ecosystems that support truly safe and sustainable innovation.

## Supplementary Material



## Notes

Supplementary figures illustrating key components of digital twin frameworks, life cycle assessment impact pathways, and NanoSolveIT IATA; Supplementary table summarizing model types, with description of their related SSbD aspects, and limitations (PDF)

## Abbreviations

AD: Applicability Domain
AOP: Adverse Outcome Pathway
ADME: Absorption, Distribution, Metabolism, and Excretion
APIs: Active Pharmaceuticals Ingredients
AUC: Area Under the Curve
AoP: Areas of Protection
AI: Artificial Intelligence
AutoML: Automated Machine Learning
BNN: Bayesian Network Modeling
BED: Biologically Effective Dose
BMD: Benchmark Dose
BMDL: Lower Bound Benchmark Dose
BMDU: Upper Bound Benchmark Dose
BMR: Benchmark Response
CNM: Carbon Nanomaterial
CF: Characterization Factor
CADRE: Computer-Aided Discovery and REdesign
DALYs: Disability-Adjusted Life Years
DEG: Differentially Expressed Gene
DFT: Density Functional Theory
EC JRC: European Commission’s Joint Research Centre
ECHA: European Chemical Agency
EF: Effect Factor
EFSA: European Food Safety Agency
EPAA: European Partnership for Alternative Approaches to Animal Testing
PARC: European Partnership for the Assessment of Risks from Chemicals
EU: European Union
ENM: Engineered Nanomaterial
FAIR: Findability, Accessibility, Interoperability, and Reusability
FAIR4RS: FAIR for Research Software
FF: Fate Factor
GHS: Global Harmonization System
HCA: hierarchical clustering analysis
HED: Human Equivalent Dose
HTS: High Throughput Screening
IATA: Integrated Approach to Testing and Assessment
IOP: Integrated Outcome Pathway
IL: Interleukin
KE: Key Biological Events
LCA: Life Cycle Assessment
LCC: Life Cycle Costing
LCI: Life Cycle Inventory
LCIA: Life Cycle Impact Assessment
*K*_*lipw*_: Liposome-Water Partition Coefficient
*D*_*lipw* ^(*pH*)^_: speciation-corrected liposome-water distribution ratio
LLM: Large Language Model
ML: Machine Learning
Cmax: Maximum Concentration
MT: Metallothionein
MoA: Mode of Action
MIE: Molecular Initiating Event
MC: Monte Carlo
MD: Molecular Dynamics
MWCNT: Multi-Walled Carbon Nanotubes
nano-QSAR: Nanomaterial Quantitative Structure Activity Relationship
nano-SAR: Nanoscale Structure–Activity Relationship
NAM: New Approach Methodology
NIOSH: National Institute for Occupational Safety and Health
NOAEL: No-Observed-Adverse Effect Level
log D_o/w_: Octanol–Water Partition Coefficient
ODE: Ordinary Differential Equations
OECD: Organisation for Economic Cooperation and Development
PBK: Physiologically-Based Kinetics
PBT: Persistence, Bioaccumulation, and Toxicity
PFAS: Per- and Polyfluoroalkyl substances
PFOS: Perfluorooctanesulfonate
PFOA: Perfluorooctanoic Acid
PMF: Free energy
PPARδ: Peroxisome Proliferator-Activated Receptor Delta
PoD: Point of Departure
PEF: Product Environmental Footprint
PEC: Predicted Environmental Concentration
TDI: Tolerable Daily Intake
