# Supplementary material for: Review on Predictive Models and Integration Strategies for Holistic Impact Assessment of Chemicals and Materials
Source: Environ Sci Technol. 2026 Jan 28;60(5):3739–66. doi: 10.1021/acs.est.5c04489 (PMC12895535; doi:10.1021/acs.est.5c04489)
Supplement: Supplementary file 1 [file es5c04489_si_001.pdf]

Review on Predictive Models and Integration Strategies for Holistic Impact Assessment of Chemicals and Materials

Angela Serra[1,2,*], Marcella Torres Maia[1], Periklis Tsiros[3], Vasileios Minadakis[3], Rafael Riudavets-Puig[4], Adrien Perello-y-bestard[5], Fotini Nikiforou[6,7], Achilleas Karakoltzidis[6,7], Emanuele Di Lieto[1], Alexandra Schaffert[1], Zeyad Al-Abdulraheem[1], Ishita Virmani[8], Olga Dziubaniuk[9], Sikri Karhukorpi[9], Joahim Dokler[10], Dimitrios Zouraris[11,12], Dimitris G. Mintis[11,12], Dimitra-Danai Varsou[11,13], Andreas Tsoumanis[12,13], Georgia Melagraki[14], Panagiotis Isigonis[15], Anastasios G. Papadiamantis[11,12,13], Marija Buljan[4], Anna Agalliadou[6,7], Laura-Jayne A. Ellis[18,19], Jacques–Aurélien Sergent[20], Matheus Alves Siqueira de Assunção[21], Diego Stéfani Teodoro Martinez[21], David Winkler[22,23,24], Seung-Geun Park[25], Seung Min Ha[25], Zayakhuu Gerelkhuu[26], Tae Hyun Yoon[25,26], Spyros Karakitsios[6,7], Dimosthenis A. Sarigiannis[6,7], Antreas Afantitis[11,12,13], Stefano Cucurachi[5], Tommaso Serchi[15], Antonino Marvuglia[15], Thomas Exner[10], Jaakko Siltaloppi[9], Martin Paparella[8], Willie Peijnenburg[16,17], Peter Wick[4], Iseult Lynch[18,19], Haralambos Sarimveis[3], Dario Greco[1,2,*]

1. Finnish Hub for Development and Validation of Integrated Approaches (FHAIVE), Faculty of Medicine and Health Technology, Tampere University, 33100 Tampere, Finland
2. Division of Pharmaceutical Biosciences, Faculty of Pharmacy, University of Helsinki, Helsinki 00790, Finland
3. National Technical University of Athens, Zografou 15772, Greece
4. Swiss Federal Laboratories for Materials Science and Technology, Lerchenfeldstrasse 5, CH-9014 St. Gallen, Switzerland
5. Institute of Environmental Sciences, Leiden University, P.O. Box 9518, 2300 RA Leiden, the Netherlands
6. Aristotle University of Thessaloniki, Department of Chemical Engineering, Environmental Engineering Laboratory, University Campus, Thessaloniki 54124, Greece
7. HERACLES Research Center on the Exposome and Health, Center for Interdisciplinary Research and Innovation, Balkan Center, Bldg. B, 10th km Thessaloniki – Thermi Road, 57001, Greece
8. Institute of Medical Biochemistry, Medical University Innsbruck, Innrain 80, 6020, Austria
9. Tampere University, Faculty of Management and Business, Industrial Engineering and Management Unit, PO Box 553, 33014 Tampere University, Finland
10. Seven Past Nine d.o.o., Hribljane 10, 1380 Cerknica, Slovenia
11. Entelos Institute, Nicosia 2102, Cyprus
12. NovaMechanics Ltd, Nicosia 1070, Cyprus
13. NovaMechanics MIKE, Piraeus 18545, Greece
14. Division of Physical Sciences and Applications, Hellenic Military Academy, 16672 Vari, Greece
15. Luxembourg Institute of Science and Technology (LIST), Luxembourg
16. Institute of Environmental Sciences, Leiden University, P.O. Box 9518, 2300 RA Leiden, the Netherlands

17. National Institute for Public Health and the Environment (RIVM), Center for Safety Assessment of Substances and Products, Bilthoven, the Netherlands
18. School of Geography, Earth, and Environmental Sciences, University of Birmingham, B15 2TT, UK
19. Centre for Environmental Research and Justice, University of Birmingham, Edgbaston, B15 2TT Birmingham, United Kingdom
20. Solvay SA, Toxicological and Environmental Risk Assessment Unit, Rue de Ransbeek 310, 1120 Bruxelles, Belgium
21. Brazilian Nanotechnology National Laboratory (LNNano), Brazilian Center for Research in Energy and Materials (CNPEM), Campinas, Sao Paulo, Brazil
22. Department of Biochemistry and Chemistry, La Trobe Institute for Molecular Science, La Trobe University, Bundoora, Victoria 3086, Australia
23. Monash Institute of Pharmaceutical Sciences, Monash University, Parkville, Victoria 3052, Australia
24. School of Pharmacy, University of Nottingham, Nottingham NG7 2RD, United Kingdom
25. Department of Chemistry, College of Natural Sciences, Hanyang University, Seoul 04763, Korea
26. Institute of Next Generation Material Design, Hanyang University, Seoul 04763, Korea

* Email: dario.greco@tuni.fi, angela.serra@tuni.fi

SUPPLEMENTARY FIGURES AND TABLES

Figure S1. Key components of a proposed framework for the development of a digital twin for material systems. **Page S4**

Figure S2. A simplified schema of how genomic descriptors (red boxes) within the Phenopacket schema[1] can be connected to single cell transcriptomic data and models to develop digital twins of organ function. **Page S5**

Figure S3. Schematic and simplified representation of the link between emissions and resources, midpoint impact categories, and damage-oriented endpoint categories in LCA. **Page S6**

Figure S4. Tier 1 of the NanoSolveIT in silico IATA focused on integrating models to predict human or environmental exposure to nanomaterials. **Page S7**

Table S1: Summary of model types, the SSbD aspects and characteristics they address and their potential limitations. **Page S8**

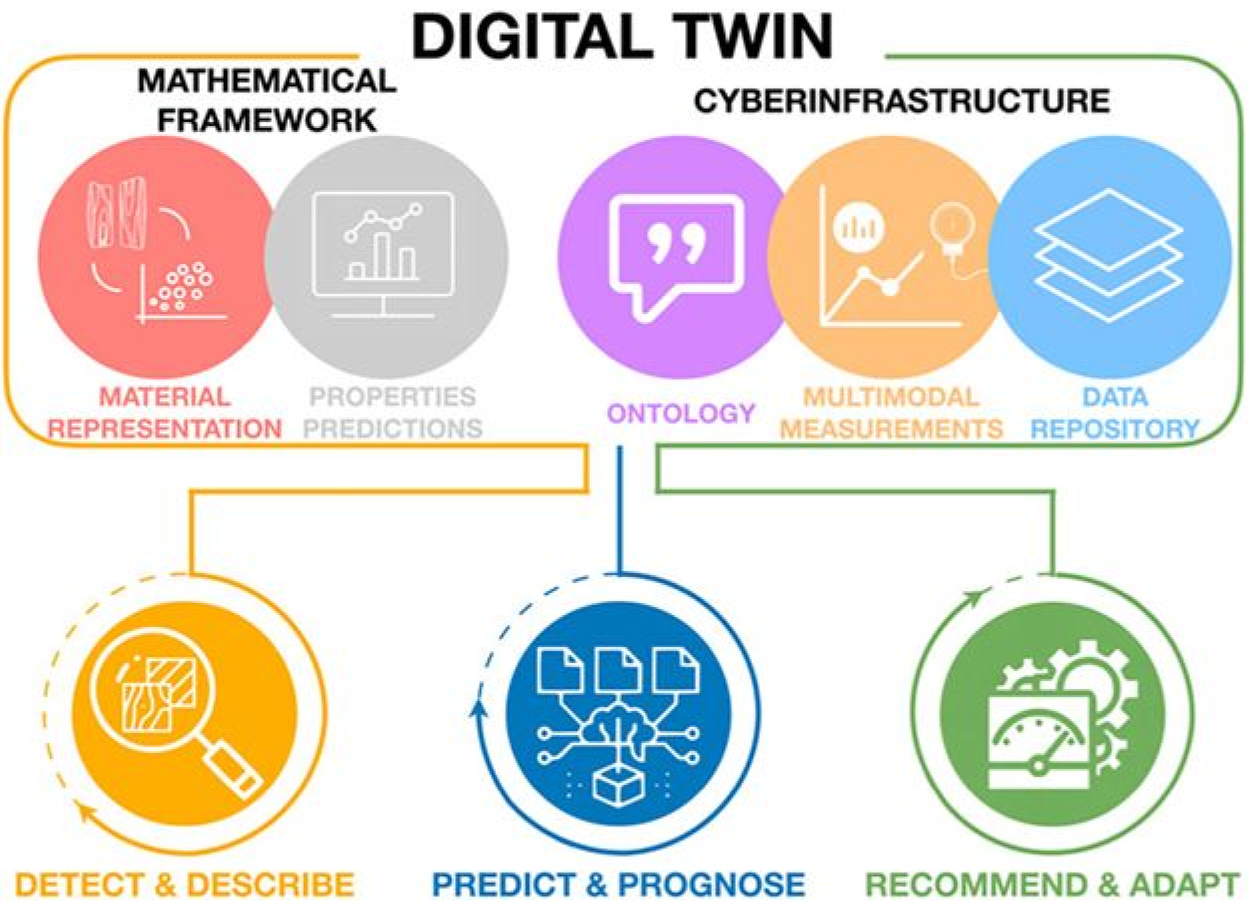

Figure S1. Key components of a proposed framework for the development of a digital twin for material systems. Reproduced from [2]. Available under a CC-BY 4.0 license. Copyright 2022 Frontiers, Kalidindi, Buzzy, Boyce and Dingreville.

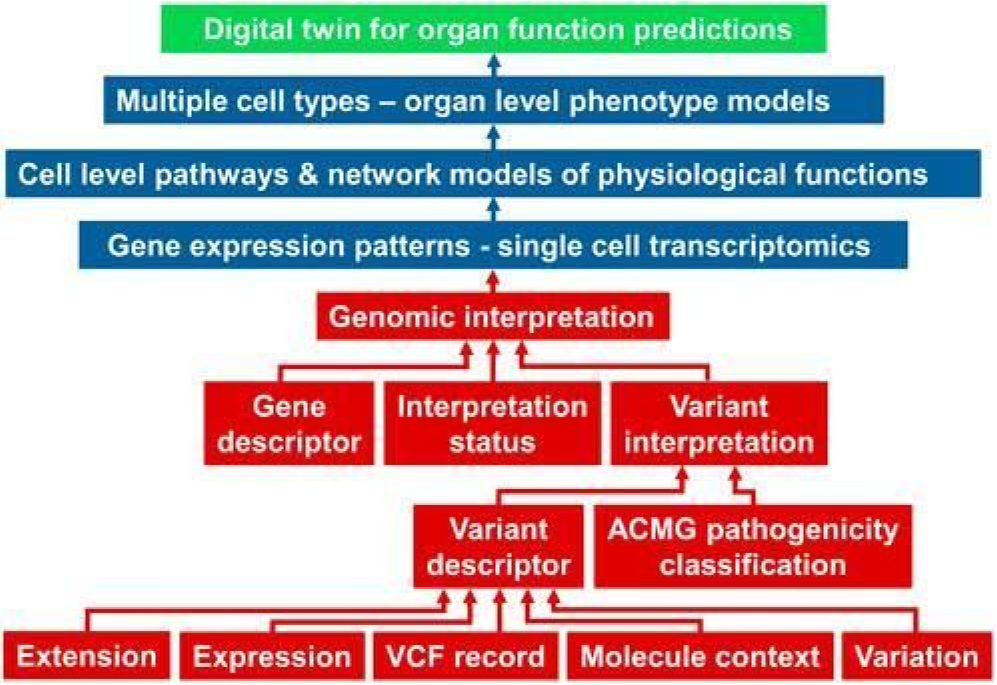

Figure S2. A simplified schema of how genomic descriptors (red boxes) within the Phenopacket schema[1] can be connected to single cell transcriptomic data and models to develop digital twins of organ function. ACMG, American College of Medical Genetics; VCF, variant call format. Reproduced from [3]. Available under a CC-BY 4.0 license. Copyright 2024 Frontiers, Hansen, Jain, Nenov, Robinson and Iyengar.

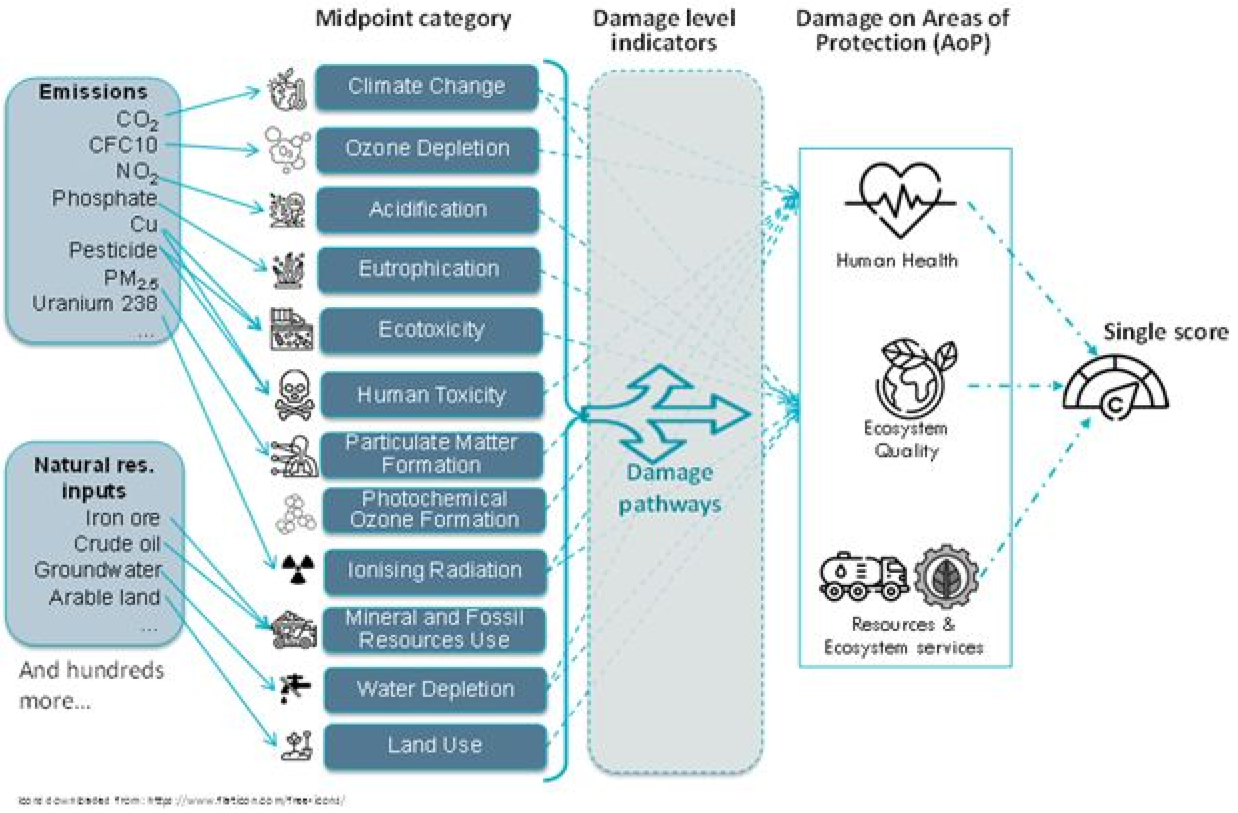

Figure S3. Schematic and simplified representation of the link between emissions and resources, midpoint impact categories, and damage-oriented endpoint categories in LCA. Midpoint level indicators refer to pollutant emission or resource use. Damage level indicators refer to the damage produced by pollutant emissions and use of resources. The Areas of Protection (AoP) refers to human health; ecosystem quality, and resources (that includes ecosystem services). The impacts on the AoP can be further aggregated using a normalisation and weighting process.

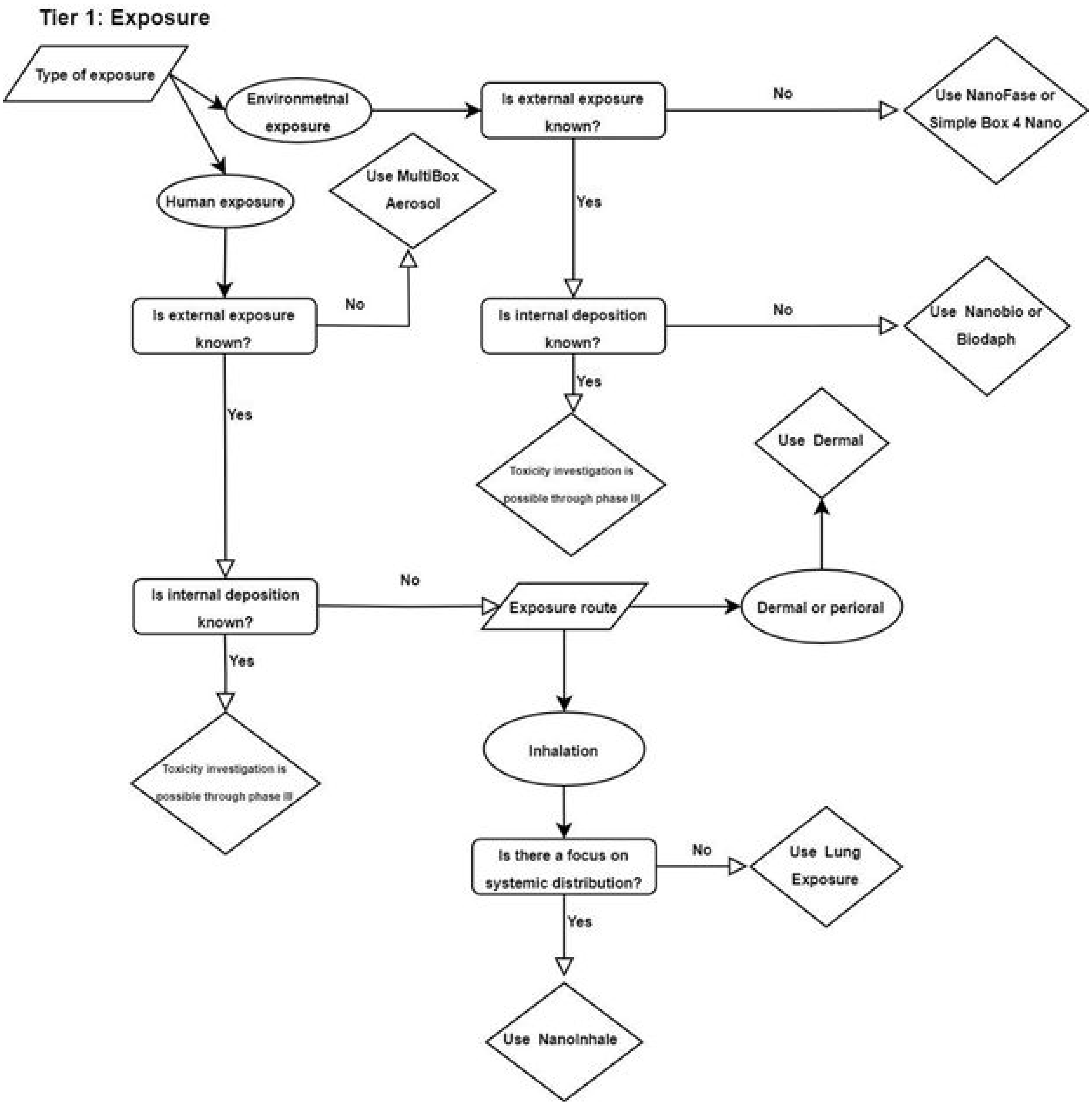

Figure S4. Tier 1 of the NanoSolveIT in silico IATA focused on integrating models to predict human or environmental exposure to nanomaterials. The models that constitute Tier 1 of the IATA are all available as web applications, at the following links: NanoFASE Soil-Water-Air model{C (https://nanofase.cloud.nanosolveit.eu/); Simple Box 4 Nano (https://sb4n.cloud.nanosolveit.eu/); MultiBox Aerosol (https://aerosol.cloud.nanosolveit.eu/); NanoBio PBPK (https://nanobio.cloud.nanosolveit.eu/): BioDaph PBPK (https://biodaph.cloud.nanosolveit.eu/); Dermal uptake (https://dermal.cloud.nanosolveit.eu/); Lung Exposure (https://lungexposure.cloud.nanosolveit.eu/); and NanoInhale (https://nanoinhale.cloud.nanosolveit.eu/).

Table S1: Summary of model types, the SSbD aspects and characteristics they address and their potential limitations.

**Model type**	**SSbD aspects/characteristics**	**Limitations**
AOP-based models	Safety - risk assessment (decision support), cross-species hazard extrapolation	Biological coverage gaps: The currently available AOPs do not yet cover all SSbD-relevant outcomes and species/life stages<br><br>Context transferability and integration uncertainty: Mapping diverse NAM outputs to AOP KEs across different experimental scenarios is uncertain, and methods for uncertainty propagation and harmonised multi-NAM integration into AOP-based IATAs are still immature<br><br>Limited quantitative readiness of AOPs: Most AOPs remain qualitative, require diverse multi-level data, and are therefore less suitable for early development where such data are lacking.
QSAR	Safety - hazard predictions (e.g., carcinogenicity, mutagenicity), property prediction (e.g., physicochemical properties)	Data quality and curation needed, limited applicability domain (e.g., limited to polymers, complex mixtures), activity cliffs.

Toxicokinetic models - PBK	Safety - risk assessment (exposure translation to target organ dose)	Extensive data requirements, with frequent data gaps, often limited concentration data available for validation, and parameter uncertainty
Toxicogenomics-based models	Safety - hazard identification & PoD derivation	Might not be suitable for early innovation stages; these models require data (i.e., physicochemical or biological data) that are often not available at early stages of the innovation process (e.g., when a new substance is designed).<br><br>Biological relevance (biological system-dependent response) and human translatability: Cellular responses vary across models (e.g., cell types, organoids, in vitro vs in vivo), and extrapolation to humans remains challenging<br><br>Limited maturity for decision-support: While SSbD explicitly allows the use of non-regulatory-accepted NAMs as a pre-market approach, TGx-based methods are still being standardized and tested, and may not yet provide limit values that are readily comparable to established endpoints within the SSbD framework, thereby

		constraining their immediate use in consistent assessment processes.
Dose-response models	Safety - risk assessment (PoD estimation)	Might not be suitable for early innovation stages<br><br>Data and endpoint alignment: Require high-quality, multi-dose datasets, which are often unavailable in early innovation, and their outputs are not yet well aligned with established SSbD endpoints, limiting comparability and decision making.
Read-across & grouping	Safety - hazard predictions (e.g., carcinogenicity, mutagenicity), property prediction (e.g., physicochemical properties)*	Lack of standardization: Criteria for analogue selection, data integration, and uncertainty assessment are not harmonized, leading to variable confidence and limited comparability of read-across results in SSbD.<br><br>Limited data availability: Suitable, high-quality reference substances are often lacking, especially for novel, complex, or UVCB materials, restricting the applicability of read-across in early SSbD stages.
Digital Twin approaches	Safety - (early) hazard and exposure assessment, process	Data quality: Incomplete or noisy data can introduce uncertainty, especially in

	optimization for manufacturing purposes<br><br>Sustainability - digital LCA estimation and circularity	safety-critical applications where accurate materials property data is essential<br><br>Need for the adoption of common data standards and models to perform consistent safety or sustainability assessments
Exposure models	Safety - occupational exposure (exposure estimation of workers during the production process)	Limited application for complex substances (e.g., UVCBs, ENMs, polymers) and exposure routes (oral, dermal, inhalation)
	Safety - consumer exposure (exposure estimation from consumer products)	
	Safety - environmental exposure & fate (exposure estimation in the different environmental media (air, water, soil))	
LCA based models/LCA software	Sustainability - evaluation of environmental impacts throughout the entire life cycle of a chemical, material, process or product	Lack of Life Cycle Inventory data and characterization factors can hinder the analysis.<br><br>LCI: data is typically withheld by companies (usually for economic/legal concerns)<br><br>CFs: lack of robust characterization factors/models to assess the toxicity of complex

		substances, such as PFAS and ENMs (e.g., fate, exposure, toxicity PODs)
S-LCA based models	Sustainability - evaluation of social impacts of products or services through their whole life cycle	Lack of life cycle inventory data can hinder analysis.<br><br>Dependence on context-specific inventory and socio-economic performance data for accurate results, which may be withheld by companies.<br><br>Utilization of data based on sectoral / global averages with existing tools.<br><br>Lack of standardization on the methods to be used. Lack of models and characterization factors for causal impact analysis.

*Difference with QSAR: read-across & grouping are expert-driven, based on fewer substances

References

(1) Danis, D.; Jacobsen, J. O. B.; Wagner, A. H.; Groza, T.; Beckwith, M. A.; Rekerle, L.; Carmody, L. C.; Reese, J.; Hegde, H.; Ladewig, M. S.; Seitz, B.; Munoz-Torres, M.; Harris, N. L.; Rambla, J.; Baudis, M.; Mungall, C. J.; Haendel, M. A.; Robinson, P. N. Phenopacket-Tools: Building and Validating GA4GH Phenopackets. *PLOS ONE* **2023**, *18* (5), e0285433. https://doi.org/10.1371/journal.pone.0285433.

(2) Kalidindi, S. R.; Buzzy, M.; Boyce, B. L.; Dingreville, R. Digital Twins for Materials. *Front. Mater.* **2022**, *9*. https://doi.org/10.3389/fmats.2022.818535.

(3) Hansen, J.; Jain, A. R.; Nenov, P.; Robinson, P. N.; Iyengar, R. From Transcriptomics to Digital Twins of Organ Function. *Front. Cell Dev. Biol.* **2024**, *12*. https://doi.org/10.3389/fcell.2024.1240384.
